# Identification of factors associated with opioid-related and hepatitis C virus-related hospitalisations at the ZIP code area level in the USA: an ecological and modelling study

**DOI:** 10.1016/S2468-2667(24)00076-8

**Published:** 2024-06

**Authors:** Fatih Gezer, Kerry A Howard, Alain H Litwin, Natasha K Martin, Lior Rennert

**Affiliations:** **Department of Public Health Sciences** (F Gezer PhD, K A Howard PhD, L Rennert PhD), **Center for Public Health Modeling and Response** (F Gezer, K A Howard, L Rennert), **and Clemson University School of Health Research** (Prof A H Litwin MD), **Clemson University, Clemson, SC, USA; Prisma Health-Upstate, Greenville, SC, USA** (Prof A H Litwin); **University of South Carolina School of Medicine Greenville, Greenville, SC, USA** (Prof A H Litwin); **Division of Infectious Disease and Global Public Health, School of Medicine, University of California San Diego, San Diego, CA, USA** (Prof N K Martin PhD)

## Abstract

**Background:**

Opioid overdose and related diseases remain a growing public health crisis in the USA. Identifying sociostructural and other contextual factors associated with adverse health outcomes is needed to improve prediction models to inform policy and interventions. We aimed to identify high-risk communities for targeted delivery of screening and prevention interventions for opioid use disorder and hepatitis C virus (HCV).

**Methods:**

In this ecological and modelling study, we fit mixed-effects negative binomial regression models to identify factors associated with, and predict, opioid-related and HCV-related hospitalisations for ZIP code tabulation areas (ZCTAs) in South Carolina, USA. All individuals aged 18 years or older living in South Carolina from Jan 1, 2016, to Dec 31, 2021, were included. Data on opioid-related and HCV-related hospitalisations, as well as data on additional individual-level variables, were collected from medical claims records, which were obtained from the South Carolina Revenue and Fiscal Affairs Office. Demographic and socioeconomic variables were obtained from the United States Census Bureau (American Community Survey, 2021) with additional structural health-care barrier data obtained from South Carolina’s Center for Rural and Primary Health Care, and the American Hospital Directory.

**Findings:**

Between Jan 1, 2016, and Dec 31, 2021, 41 691 individuals were hospitalised for opioid misuse and 26 860 were hospitalised for HCV. There were a median of 80 (IQR 24–213) opioid-related hospitalisations and 61 (21–196) HCV-related hospitalisations per ZCTA. A standard deviation increase in ZCTA-level uninsured rate (relative risk 1·24 [95% CI 1·17–1·31]), poverty rate (1·24 [1·17–1·31]), mortality (1·18 [1·12–1·25]), and social vulnerability index (1·17 [1·10–1·24]) was significantly associated with increased combined opioid-related and HCV-related hospitalisation rates. A standard deviation increase in ZCTA-level income (0·79 [0·75–0·84]) and unemployment rate (0·87 [0·82–0·93]) was significantly associated with decreased combined opioid-related and HCV-related hospitalisations. Using 2016–20 hospitalisations as training data, our models predicted ZCTA-level opioid-related hospitalisations in 2021 with a median of 80·4% (IQR 66·8–91·1) accuracy and HCV-related hospitalisations in 2021 with a median of 75·2% (61·2–87·7) accuracy. Several underserved high-risk ZCTAs were identified for delivery of targeted interventions.

**Interpretation:**

Our results suggest that individuals from economically disadvantaged and medically under-resourced communities are more likely to have an opioid-related or HCV-related hospitalisation. In conjunction with hospitalisation forecasts, our results could be used to identify and prioritise high-risk, underserved communities for delivery of field-level interventions.

**Funding:**

South Carolina Center for Rural and Primary Healthcare, National Institute on Drug Abuse, and National Library of Medicine.

## Introduction

Recent escalation in the number of opioid-related hospitalisations and deaths has raised concerns about the prevalence of opioid misuse and its effect on public health in the USA.^1,2^ Annual deaths caused by overdose have increased by more than 50% since January, 2020, surpassing 110 000 in 2023.^3^ Additionally, opioid use through injection drug practices is creating a surge in infectious disease prevalence, adding to the public health crisis.^1^ Notably, the number of diagnosed hepatitis C virus (HCV) infections doubled between 2013 and 2020,^4^ and has continued to increase since 2020, presumably owing to the opioid epidemic.^5^ As such, health-care professionals and policy makers have prioritised efforts to prevent and treat the misuse of opioids and HCV. However, treatment uptake among populations with opioid use disorder and HCV infection are low, with only about 25% of those with opioid use disorder and 15% of those with HCV receiving treatment.^6^ Efforts to facilitate access to resources and increase uptake are key to alleviating the public health crisis.^7^ Targeted programming to areas with populations at high risk of opioid misuse and HCV infection can create effective and efficient allocation of lifesaving resources and funding to appropriate US states and ZIP codes.^8^ Therefore, understanding predictive factors of hospitalisations for opioids and HCV can improve treatment and prevention efforts.

The incidence of opioid-related and HCV-related adverse events varies according to geographical factors.^9^ Previous studies have investigated patterns of opioid use, such as variations based on neighbourhoods, cities, and rural regions.^10–12^ Several studies have focused on prediction of opioid-related events at the county level using statistical models based on sociodemographic factors, access to health-care facilities, and drug markets.^13–15^ Studies examining opioid-related events at the local community level are crucial for informing policy makers and optimising interventions.^16^ Sociostructural circumstances vary across communities based on their unique historical, geographical, and cultural background, and, therefore, tailored approaches specific to locations are required.^16,17^

Although one 2023 study focused on prediction of opioid-related outcomes (ie, mortality) at geographically granular levels (eg, ZIP code),^18^ it did not identify factors associated with mortality. A few studies examined the role of demographic and socioeconomic factors associated with HCV at the ZIP code level but did not implement predictive modelling.^19,20^ Another study identified hotspots for HCV and drug overdose cases among individuals using heroin and cocaine participating in a substance use treatment programme and was not representative of the general at-risk population.^21^ To our knowledge, there are no studies integrating predictive models with public health response (eg, intervention implementation) for opioid use disorder and HCV.

Mobile health clinics are effective interventions for providing health care to populations with access barriers.^22^ The South Carolina Center for Rural and Primary Healthcare (SCCRPH), tasked by the state government to assist with coordinating mobile health clinic operations, and the National Institute on Drug Abuse, funded this study to help identify and prioritise high-risk rural and underserved communities for opioid and HCV screening, treatment, and prevention interventions via mobile health clinics. These efforts require localised predictions to pinpoint where to send mobile health clinics.

The objectives of this study were to identify local-level community factors associated with opioid-related and HCV-related hospitalisations and to predict opioid-related and HCV-related hospitalisations at the ZIP code tabulation area (ZCTA) level. The first objective is important for identifying potentially modifiable factors (eg, barriers to health-care access) and tailoring educational and intervention efforts. The second objective is useful for identifying high-risk communities for intervention placement. Information on factors associated with adverse opioid or HCV outcomes could be used for strategic community outreach to promote the use of mobile health clinics or tailor interventions to sustain opioid treatment.

## Methods

### Study design and data sources

This ecological and modelling study included all adults aged 18 years and older living in South Carolina (USA) between Jan 1, 2016, and Dec 31, 2021.

All opioid-related and HCV-related hospitalisation encounters in South Carolina during this period were obtained from the South Carolina Revenue and Fiscal Affairs Office.^23^ Additional variables at the individual level were age, race, sex assigned at birth, insurance status, and home ZCTA; data for these variables were also obtained from the South Carolina Revenue and Fiscal Affairs Office.

Demographic, socioeconomic, and structural barrier data were linked to the ZCTA of each hospitalised patient. Demographic and socioeconomic variables were obtained from the United States Census Bureau (American Community Survey, 2021), and included age, sex assigned at birth, race, ethnicity, adult population size (aged 18 years or older), median household income, unemployment rate, and labour force participation rate.^24^ The social vulnerability index, provided by the Agency for Toxic Substances and Disease Registry at the Centers for Disease Control and Prevention,^25^ measures potential risks to communities caused by disasters and is calculated by four compounds: socioeconomic status; household composition and disability; minority status and language; and housing and transportation.^26^ As a percentile rank, higher values denote greater vulnerability. Data are available at each census tract. ZCTA-level social vulnerability index was calculated by averaging over census tracts for each ZCTA (weighted by census tract population size). Structural barrier variables, which serve as surrogates for health-care access, were obtained from SCCRPH.^27^ For each ZCTA, these variables were hospital presence (yes or no), primary care physicians and medical doctors or doctors of osteopathic medicine per 1000 residents, and proportion of individuals who are uninsured. Data on hospital size were collected through the American Hospital Directory. Remaining variables (obtained from SCCRPH) were all-cause mortality per 1000 residents in each ZCTA and the proportion of ZCTAs covered by rural areas.

The demographic and socioeconomic covariates in these models were selected on the basis of similar studies in the literature and data availability.^13,18,28^ Structural barriers to health-care access were chosen on the basis of consultation with relevant stakeholders from Clemson Rural Health and SCCRPH.

The outcomes of interest were aggregated opioid-related and HCV-related patient hospitalisations at the ZCTA level for each period (ie, quarter or year). ICD-10 codes for classification of opioid-related and HCV-related hospitalisations are in the appendix (pp 1–2). We also considered the sum of opioid and HCV (ie, combined) hospitalisations.

Ethics review for this study was obtained from the Institutional Review Board of Clemson University (number 2020–0150). The Institutional Review Board of Clemson University determined that the data and analysis plan met the exemption criteria under federal regulations at 45 CFR 46, wherefore no informed consent was required.

### Statistical analysis

We used negative binomial generalised linear mixed models to examine the association between the annual hospitalisation rate in each ZCTA and demographic, socioeconomic, and structural barrier variables (objective 1). Individual-level variables could not be modelled owing to the absence of covariate information for those without a hospitalisation. Number of hospitalisations refers to the number of unique individuals hospitalised each year. In the partially adjusted models, each ZCTA-level predictor was fit independently, adjusting for year (linear) and ZCTA-level random intercepts and slopes for year. As a sensitivity analysis, we also adjusted for hospital presence and hospital size (measured through number of available patient beds)^29^ to account for potential confounding of the effect of community-level factors on hospitalisations. Additional sensitivity analysis included interrupted time series for negative binomial generalised linear mixed model to account for the COVID-19 effect. p values less than 0·05 (two-sided) were considered statistically significant.

Fully adjusted models were used to predict opioid-related and HCV-related hospitalisations (objective 2). Separate models were fit to predict hospitalisation rates at both quarterly and annual time periods. Prediction at the quarterly level is useful for mobile health clinic allocation, as these clinics can spend 3 months at the same site. However, annual predictions were also calculated due to higher prediction accuracy and their usefulness for other community-based interventions, which typically have longer implementation windows. These prediction models were also adjusted for lagged hospitalisation count (models for HCV prediction also included the lagged cumulative opioid hospitalisation count).

To evaluate the accuracy of the models, we separated the data into training and validation sets. The first training set consisted of all data between Jan 1, 2016, and Dec 31, 2020, and the validation set consisted of all data from Jan 1 to Dec 31, 2021. We did the analysis using quarterly data (validation periods: quarters 1, 2, 3, and 4 of 2021) and yearly data (validation period: 2021). To evaluate prediction performance in practice where reported hospitalisations were lagged by 1 or more years,^16^ these predictions were repeated using a second training set consisting of all data between Jan 1, 2016, and Dec 31, 2019, and using 2021 data for validation. We compared the model performances on the basis of various metrics, which were median agreement percentage, median absolute error, root mean squared error, and ranking agreement percentage (appendix p 3). To assess sensitivity to model structure, we conducted sensitivity analyses using a Poisson generalised linear mixed model and conditional autoregression model (to accommodate spatiotemporal effects).^18^ To examine the effect of including covariates in predictive performance, we compared fully adjusted models with their partially adjusted counterparts (adjusting for previous hospitalisation, time, and random effects only) and to the predictive performance of previous year hospitalisations only. We predicted 2023 hospitalisations (training set: 2016–21 data) to identify the highest risk, medically underserved ZCTAs for prioritisation of mobile health clinic delivery. We restricted our focus to the upstate and midlands regions of South Carolina because they are within the jurisdiction of Clemson Rural Health, our mobile health clinic implementation partner.^30^ All model details are in the appendix (pp 4–5). We used R (version 4.3.1) programming language for all analyses conducted in this research.

### Role of the funding source

The funders of the study had no role in study design, data collection, data analysis, data interpretation, or writing of the report.

## Results

Between Jan 1, 2016, and Dec 31, 2021, in South Carolina, 41 691 individuals were hospitalised for opioid misuse and 26 860 were hospitalised for HCV. The median number of annual opioid-related hospitalisations per individual was 1 (IQR 1–2) and the median number of annual HCV-related hospitalisations per individual was 2 (1–3). 5544 individuals were hospitalised for both causes, constituting 13·3% of opioid hospitalisations and 20·6% of HCV hospitalisations.

Relative to the median age of the population of South Carolina and individuals with an opioid-related hospitalisation, individuals with an HCV-related hospitalisation were older (table 1). There were fewer male than female individuals in the population of South Carolina; however, there were more male individuals than female individuals with opioid-related hospitalisation and HCV-related hospitalisation (table 1). Compared with the statewide population, there was a higher proportion of White individuals with opioid-related hospitalisations and HCV-related hospitalisations (table 1). Relative to the statewide population, there was a lower proportion of Black individuals with opioid-related hospitalisations but a similar proportion with HCV-related hospitalisations (table 1). For both hospitalisation types, the proportion of Hispanic individuals was substantially lower than in the statewide population. The proportion of uninsured individuals was greater for both hospitalisation types compared with the statewide population (table 1).

There were modest changes in patient demographics during the study period (appendix pp 6–7). For opioid-related and HCV-related hospitalisations, we observed slight increases in the proportion of individuals aged 30–44 years, aged 65 years and older, who were male, and who were uninsured. Notably, for HCV-related hospitalisations, there was a large decrease in the proportion of individuals aged 45–64 years.

Between 2016 and 2021, there were a median of 80 (IQR 24–213) and a mean of 151 (SD 196) opioid-related hospitalisations per ZCTA, a median of 61 (IQR 21–196) and a mean of 132 (SD 165) HCV-related hospitalisations per ZCTA, and a median of 130 (IQR 44–392) and a mean of 275 (SD 336) combined hospitalisations per ZCTA. Summary statistics for ZCTA-level community characteristics and hospitalisations are in the appendix (pp 6–7).

Estimated relative risk (RR) with associated 95% CIs for the association between socioeconomic and healthcare access variables and opioid-related and HCV-related hospitalisations at the ZCTA level (objective 1) are in table 2. The RR indicates the relative change in hospitalisation rate corresponding to a standard deviation increase in the predictor variable (unless otherwise indicated), at a fixed timepoint for a typical ZCTA.

At the ZCTA level, characteristics significantly associated with increased opioid-related and HCV-related hospitalisations included lower median income, higher uninsured rate, higher social vulnerability index, higher poverty rate, lower unemployed rate, and higher mortality (table 2). Greater labour force participation was associated with higher opioid-related hospitalisations but lower HCV-related hospitalisations. Greater numbers of primary care physicians, medical doctors, and doctors of osteopathic medicine were associated with higher HCV-related hospitalisations. Opioid-related hospitalisations were higher in rural areas. Both opioid-related and HCV-related hospitalisations decreased each year (by 1·0% and 4·7%, respectively).

ZCTAs with a higher proportion of individuals aged 45–64 years were more likely to have higher opioid-related and HCV-related hospitalisations (table 2). ZCTAs with a higher proportion of individuals aged 30–44 years and aged 65 years and older were associated with lower HCV-related hospitalisations. The results were similar for the sensitivity analyses adjusting for hospital presence and size, both of which were significantly associated with higher HCV-related and combined hospitalisations (appendix pp 8–9).

Fully adjusted models included all covariates with the exception of number of medical doctors or doctors of osteopathic medicine per 1000 people owing to the high collinearity with the number of primary care physicians, measured via variance inflation factor^31^ (appendix pp 10–11). The regression coefficients for fully adjusted models for prediction (objective 2) show the effect size for each variable (appendix p 12). Median ZCTA household income had the strongest effect size for both opioid-related and HCV-related hospitalisations. Random effect variances and likelihood ratio tests are in the appendix (p 13). Results for partially adjusted and fully adjusted Poisson generalised linear mixed models and conditional autoregressive models are in the appendix (pp 14–17). Our sensitivity analyses using interrupted time series models did not find evidence that the COVID-19 pandemic had a significant impact on study findings (appendix pp 21–23).

The median percent agreements of predictions for various validation data and model scenarios are shown in table 3. Prediction accuracies based on the fully adjusted negative binomial generalised linear mixed model were median 80·4% (IQR 66·8–91·1) for opioid-related hospitalisations, 75·2% (61·2–87·7) for HCV-related hospitalisations, and 84·2% (71·5–92·0) for combined hospitalisations using 2016–20 data, all of which were greater than the prediction accuracies using previous ZCTA-level hospitalisations only. Accuracy based on a 1-year lag was similar (model scenario 2 in table 3). There was a decrease in model accuracy for quarterly-level predictions compared with annual predictions. Similar trends were observed using additional agreement metrics (appendix pp 18–20).

Factors associated with hospitalisations between the generalised linear mixed model and conditional auto-regressive model were similar (appendix pp 14, 16); however, prediction accuracy of the conditional auto-regressive model was substantially lower than that of the generalised linear mixed model (appendix pp 18–20). We also compared model performance with models including previous hospitalisations, time, and random effects only. Although the negative binomial generalised linear mixed model was the best performing, there was only a mild increase in prediction accuracy through inclusion of community-level covariates (appendix pp 18–20).

The predicted opioid-related, HCV-related, and combined hospitalisations in South Carolina for the 2023 calendar year (training set: 2016–21) are shown in figure A–C (reference map of South Carolina counties shown in appendix p 24). In 2023, the total number of predicted opioid-related hospitalisations was 8912, HCV-related hospitalisations was 6010, and combined hospitalisations was 14 716. The predicted combined hospitalisations for the 20 highest risk ZCTAs in the upstate and midlands regions of South Carolina are shown in figure D. ZCTAs 29611, 29203, and 29624 had the highest combined hospitalisations with high social vulnerability index, high proportions of uninsured people, and limited presence of hospitals and treatment facilities. The predicted opioid-related and HCV-related hospitalisations for the upstate and midlands regions are in appendix p 25. Characteristics related to the access barriers in the top 20 highest-risk ZCTAs for opioid-related, HCV-related, and combined hospitalisations are in appendix pp 26–28.

Our findings showed risk variation within county (appendix pp 29–32). Median agreement between county and ZCTA-level hospitalisation rates in 2021 were 75·3% (IQR 69·6–81·8) for opioid-related hospitalisations, 72·3% (63·0–70·4) for HCV-related hospitalisations, and 76·0% (67·9–83·6) for combined hospitalisations, showing the limitations of using county-predicted risk for informing local interventions.

## Discussion

Among low uptake of services, barriers to health-care access, and finite treatment resources, individuals with opioid misuse and HCV are priority populations for understanding risks related to adverse events for targeted intervention. We identified community-level factors associated with hospitalisations and incorporated these into predictive models that identified ZCTAs in South Carolina at high risk of opioid-related or HCV-related hospitalisations. Factors related to socioeconomic status and health-care access were strongly associated with hospitalisation risk. Although knowledge of these factors is important for identifying modifiable barriers, informing long-term structural interventions (eg, reducing uninsurance rates), developing tailored interventions to socioeconomic status,^32^ and identifying and prioritising high-risk, medically underserved communities can maximise the effectiveness of interventions. The results of the present study could directly aid the prioritisation of South Carolina’s underserved communities for tailored mobile health clinic interventions.

Our results show specific barriers for communities associated with the highest risk of opioid-related and HCV-related hospitalisations. ZCTAs characterised by higher proportions of uninsured people, higher social vulnerability index, higher poverty, lower median income, lower unemployment rates, higher all-cause mortality, and rurality were associated with greater risk of hospitalisations. Therefore, medically underserved communities are those most at risk of opioid-related and HCV-related hospitalisations.^33^ Many hospitalisations are potentially preventable given that these communities have access barriers that make it difficult to seek care until the opioid-related or HCV-related complications are serious enough to warrant hospitalisation.^34^

We found significant associations between hospital presence, medical personnel, and HCV-related hospitalisations, which might be contradictory because such medical infrastructure is, by definition, a measure of access to health care. There are several explanations for this. First, total hospitalisations in these communities might be heightened simply because hospital presence implies that hospitalisations will occur.^35^ However, even when geographical barriers to care are removed (eg, hospital presence in ZCTAs), medically underserved individuals might continue to refrain from preventive care that in turn would minimise hospitalisations.^36–38^ As evidenced by our sensitivity analyses, communities with greater socioeconomic vulnerability had poorer outcomes even after controlling for hospital presence. Moreover, these individuals face a greater risk of mortality and other adverse out-of-hospital complications because of the additive consequences from the absence of preventive care. Therefore, if hospitals are built in communities facing additional access barriers to care (eg, lack of insurance), such barriers need to be addressed to ensure uptake of services.

In addition to understanding community-level drivers of opioid-related and HCV-related health outcomes, our study incorporated these factors into a predictive modelling framework. Our approach yielded a high degree of accuracy, bolstering the reliability of our risk score assessment. By incorporating this validation step, our models provide a robust tool for identifying the ZCTAs that are at the highest risk of hospitalisations. Notably, we did not find evidence that our results were strongly impacted by the COVID-19 pandemic (appendix pp 21–23).

ZCTA-level factors, such as social vulnerability index, have been recommended as factors on which to base health resource distribution, including phased distribution of COVID-19 vaccines to geographical areas with the highest vulnerability to create equitable distribution.^39,40^ That said, to ensure that communities do not feel harmed by identification, it is important to engage with the community and people with lived experience with drug use. This should include establishing relationships with groups who can promote efforts to community members, establishing transparency and respect of diverse audiences, and allowing communities to have final approval for interventions.^22^ Such collaborations between communities and health-care decision makers might improve intervention uptake and effectiveness.

The study results are also practically applicable through identification of regions for targeted mobile health clinic interventions for opioid and HCV screening, treatment, and prevention. Using our predictive modelling framework, we identified multiple ZCTAs to prioritise in upcoming interventions due to high risk of hospitalisations, high social vulnerability index, large uninsured populations, and lack of health-care facilities. We are working to integrate these statistical predictions into the workflow of Clemson Rural Health, whose mobile health clinics provide opioid and HCV screening and treatment. These clinics have flexibility in their deployment within a county; therefore, ZCTA granularity to inform mobile health clinic placement is useful and feasible. Specifically, the ZCTA-level information could allow for identification of the highest priority areas of greatest need and then, through discussion with community partners, the clinics could be deployed to specific locations.^22^ The Clemson Rural Health mobile health clinic programme currently uses a collaborative approach, engaging community partners for deployment decisions.^30^ However, this does not necessarily mean that areas of highest need are being prioritised, a key component of effective resource allocation that is added by the modelling information provided here.

This study has limitations to consider. Our predictive models are based on South Carolina data and must be calibrated for other states. For example, in the USA, Hispanic individuals constituted 16·7% of people who died from opioid overdose in 2020,^41^ but contributed to only 0·6% of opioid-related hospitalisations in South Carolina in 2020, highlighting limitations to generalisability to other areas. Additionally, hospitalisation data are lagged by 1 year and demographic, socioeconomic, and other important contextual factors can be lagged by substantially longer. More timely data collection and reporting would improve prediction accuracy and identification of high-risk, medically underserved communities. Furthermore, the rapidly changing nature of drug use epidemics might mean that the models require continuous modifications, anywhere from data collection to model implementation.^13^ Examining trends in communities with substantial changes in overdose rates or disease incidence could help identify additional factors associated with improved outcomes or the introduction of latent factors that worsen health outcomes. This information might also help identify spatial outliers to understand which regions are doing better or worse than predicted by the models.

The increase in model predictive accuracy owing to covariate inclusion in this study and in another study done at the ZCTA level^18^ was modest and might therefore benefit from inclusion of additional covariates (eg, opioid prescription rates).^28^ A previous study of opioid overdose mortality at the county level showed moderate improvements in prediction accuracy through covariate inclusion.^13^ However, overall prediction accuracy in the current study was higher than the previous study examining opioid overdose mortality.^13^ Given the high prediction accuracy of our benchmark models (ie, no covariates), there might be less room for improvement. The higher prediction accuracy in this study, despite focusing on a more granular geographical region, might be due to the restriction of ZCTAs to a single state as opposed to all counties in the USA. In addition to incorporating sociodemographic data, efforts should continue to be made to collect and incorporate healthcare access variables into modelling frameworks, given their association with severe health outcomes. However, further understanding of the causal pathway between health-care access and hospital-related outcomes is needed.

Scarcity of data caused missing risk scores for some ZCTAs with small population counts. There were 48 ZCTAs with no opioid-related hospitalisation data, constituting 0·9% of the South Carolina population, and 71 ZCTAs with no HCV-related hospitalisation data, constituting 1·4% of the South Carolina population. Although some of these ZCTAs represent areas with low population densities (eg, forests), large institutions (eg, universities), or other commercial areas with minimal resident populations, others might have few data due to small population sizes. Therefore, caution should be taken when extrapolating this study’s findings to such regions. Additionally, the interpretation of the regression coefficients in the fully adjusted models are conditional, and hence this interpretation is convoluted due to other factors in the model and should not be interpreted as direct effects on the outcome.^42^

Hospitalisation data are not comprehensive of all adverse-related health events. Therefore, this modelling study should be replicated for opioid-related and HCV-related mortality, and naloxone administration for overdose reversal, as a sensitivity analysis to the limitations of underdiagnosis that is associated with hospitalisation outcomes. This analysis is important not only for prediction but for identification of factors associated with outcomes, since underdiagnosis might bias the estimated effect of such factors on severe health outcomes.

Because there is substantial variation in hospitalisation risk at the county level, ZCTA-level predictions are more informative for allocation of mobile health clinics and other field-level interventions as they allow for deployment closer to the population at risk. This helps to reduce transportation burdens on patients, which are a major barrier to seeking health care.^22^ However, more granular data (ie, census tract or block) could provide further improvements as disparities are still present within ZCTAs. That said, consideration of optimal site location within any geographical region is necessary to increase the likelihood that patients seek care. The next iteration of modelling frameworks should incorporate both predicted community risk and predicted intervention used to choose optimal site locations and increase care to a greater number of individuals in need. Additionally, although the goal of this modelling framework was to inform targeted interventions (eg, mobile health clinics) where shorter predictions are more useful, this framework can be extended to predict, and identify factors associated with, long-term trends for opioid use disorder and related conditions, which is an active area of research.

Adverse events associated with opioids and related diseases are a widespread and persistent public health problem and are impacted by geographical, socioeconomic, and health-care access barriers. Through ZCTA-level data, we built models that can predict and prioritise both high-risk and medically underserved areas for distribution of field-level interventions to aid in treating and preventing adverse health outcomes related to opioid misuse and HCV. The framework might be extended to other states or diseases and could serve as a toolkit for risk assessments, policies, and community-level interventions.

## Supplementary Material

1

## Figures and Tables

**Figure:**
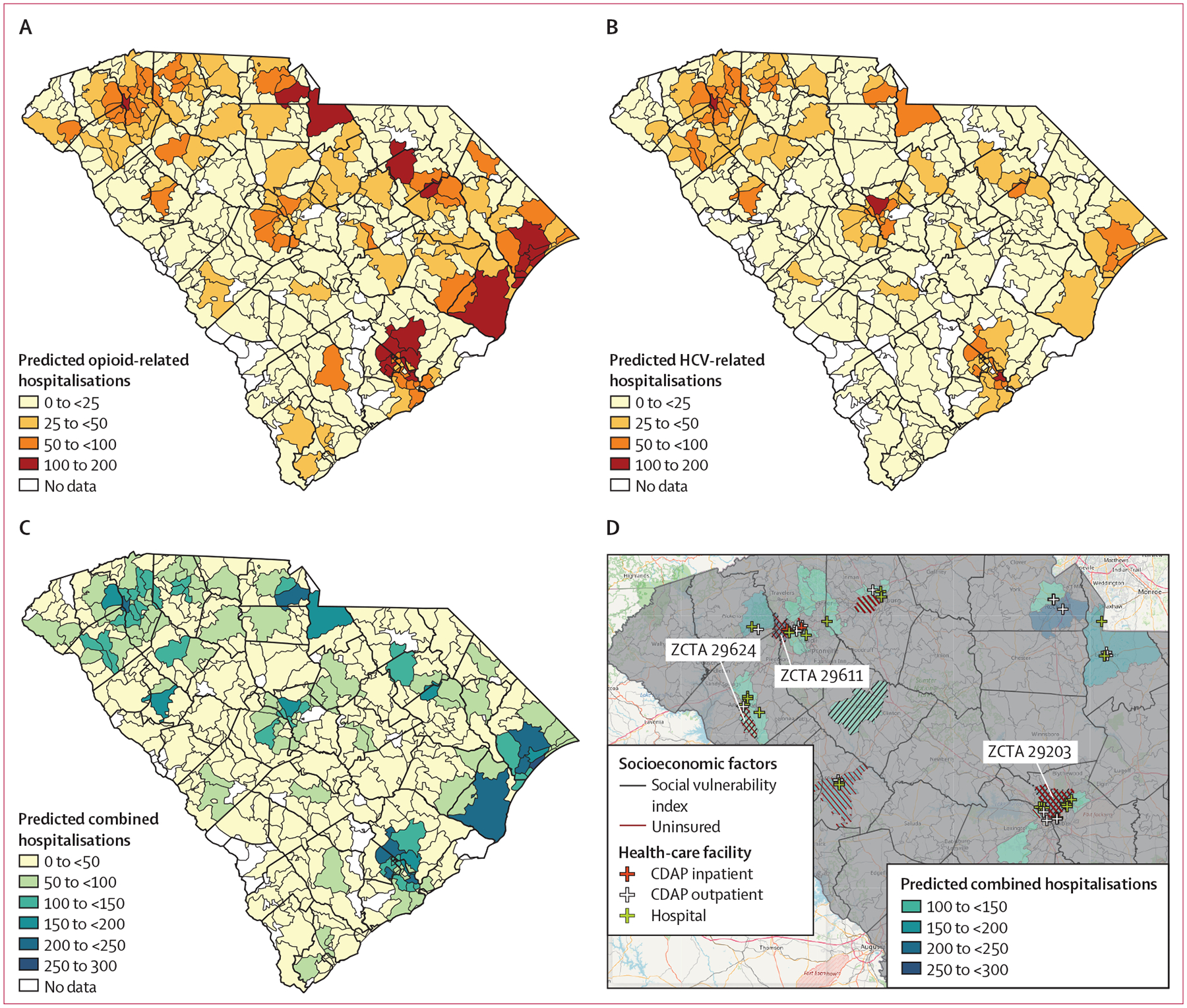
Predicted hospitalisations across ZCTAs in South Carolina, USA, in 2023 (A) Predicted opioid-related hospitalisations. (B) Predicted HCV-related hospitalisations. (C) Predicted combined hospitalisations. (D) Predicted combined hospitalisations across the top 20 highest risk ZCTAs in the upstate and midlands regions of South Carolina in 2023. Locations of the health-care facilities are marked based on the facility type. ZCTAs in the top quartile of the social vulnerability index are hatched with black lines and ZCTAs in the top quartile of the proportion of uninsured people are hatched with dark red lines. The three ZCTAs with the highest hospitalisation risk among medically underserved communities (measured by social vulnerability index, proportions of uninsured people, and access to treatment facilities) are labelled. Thick black lines represent county boundaries. CDAP=centres of drug and alcohol programmes. HCV=hepatitis C virus. ZCTA=ZIP code tabulation area.

**Table 1:** Descriptive statistics

	South Carolina population (n=5 282 634)	Opioid-related hospitalisation (n=41 691)	HCV-related hospitalisation (n=26 860)
Age, years	40*	41 (31–56)	56 (44–62)
Age group			
<18 years	1 161 158 (22·0%)	0†	0†
18–29 years	822 815 (15·6%)	9074 (21·8%)	2032 (7·6%)
30–44 years	986 354 (18·7%)	14 059 (33·7%)	4811 (17·9%)
45–64 years	1 374 646 (26·0%)	13 748 (33·0%)	15 123 (56·3%)
≥65 years	937 659 (17·7%)	4810 (11·5%)	4894 (18·2%)
Sex			
Female	2 709 991 (51·3%)	20 309 (48·7%)	10 661 (39·7%)
Male	2 572 643 (48·7%)	21 382 (51·3%)	16 199 (60·3%)
Race or ethnicity			
White	3 354 473 (63·5%)	34 665 (83·1%)	18 621 (69·3%)
Black	1 389 333 (26·3%)	5937 (14·2%)	7436 (27·7%)
Hispanic	348 654 (6·6%)	264 (0·6%)	205 (0·8%)
Other	190 174 (3·6%)	825 (2·1%)	598 (2·2%)
Insurance			
Medicaid	1 061 809 (20·1%)	9324 (22·4%)	6349 (23·6%)
Medicare	898 048 (17·0%)	10 945 (26·3%)	9111 (33·9%)
Private	2 794 514 (52·9%)	9176 (22·0%)	6419 (23·9%)
Self-pay or other	528 263 (10·0%)	12 246 (29·3%)	4981 (18·6%)

Data are median (IQR) or n (%). ICD-10 codes for hospitalisation classification are in the appendix (pp 1–2). HCV=hepatitis C virus.

* The IQR is not available because this is a singular statewide datapoint.

† Individuals younger than 18 years were not included in the study.

**Table 2:** Negative binomial generalised linear mixed effects model results for opioid-related, HCV-related, and combined hospitalisations

	Opioid-related hospitalisations (N_Hosp_=56 951, N_ZCTA_=376)		HCV-related hospitalisations (N_Hosp_=46 444, N_ZCTA_=353)		Combined hospitalisations (N_Hosp_=103 395, N_ZCTA_=376)	
	RR (95% CI)	p value	RR (95% CI)	p value	RR (95% CI)	p value
**Age (reference: proportion aged 18–29 years)**						
Proportion aged 30–44 years	1·01 (0·93–1·09)	0·82	0·89 (0·82–0·97)	0·0058	0·96 (0·89–1·04)	0·32
Proportion aged 45–64 years	1·12 (1·03–1·21)	0·0057	1·09 (1·01–1·18)	0·034	1·11 (1·03–1·20)	0·0054
Proportion aged ≥65 years	1·04 (0·96–1·13)	0·37	0·85 (0·79–0·92)	<0·0001	0·97 (0·89–1·05)	0·39
**Sex (reference: proportion female)**						
Proportion male	0·98 (0·92–1·04)	0·50	0·99 (0·91–1·08)	0·87	0·97 (0·91–1·03)	0·28
**Race (reference: proportion White)**						
Proportion Black	0·97 (0·91–1·03)	0·37	0·98 (0·92–1·05)	0·64	0·97 (0·91–1·03)	0·31
Proportion other race	0.96 (0·91–1·02)	0·22	1·01 (0·94–1·09)	0·80	0·97 (0·92–1·03)	0·39
**Ethnicity (reference: proportion non-Hispanic)**						
Proportion Hispanic	0·99 (0·93–1·06)	0·83	1·01 (0·94–1·07)	0·87	1·00 (0·94–1·07)	0·90
**Other variables**						
Years since start of study period*	0·99 (0·98–1·00)	0·017	0·95 (0·93–0·98)	0·0001	0·97 (0·96–0·98)	<0·0001
Lagged hospitalisation count	1·10 (1·06–1·13)	<0·0001	1·01 (1·00–1·03)	0·14	1·12 (1·08–1·16)	<0·0001
Cumulative lagged hospitalisation count	··	··	1·01 (1·00–1·03)	0·080	··	··
**Socioeconomic variables**						
Social vulnerability index	1·18 (1·12–1·25)	<0·0001	1·17 (1·09–1·24)	<0·0001	1·17 (1·10–1·24)	<0·0001
Median income	0·78 (0·78–0·78)	<0·0001	0·81 (0·76–0·86)	<0·0001	0·79 (0·75–0·84)	<0·0001
Proportion in poverty	1·23 (1·16–1·31)	<0·0001	1·22 (1·15–1·31)	<0·0001	1·24 (1·17–1·31)	<0·0001
Proportion unemployed	0·91 (0·85–0·97)	0·0037	0·93 (0·87–1·00)	0·037	0·87 (0·82–0·93)	<0·0001
Proportion labour force participation	1·12 (1·05–1·19)	0·0007	0·91 (0·85–0·98)	0·015	1·02 (0·95–1·08)	0·62
Proportion rural	1·11 (1·04–1·17)	0·0007	1.02 (0·96–1·09)	0·53	1·06 (1·00–1·12)	0·072
**Health-care access**						
Proportion uninsured	1·24 (1·17–1·31)	<0·0001	1·18 (1·11–1·26)	<0·0001	1·24 (1·17–1·31)	<0·0001
Number of primary care physicians per 1000 people	1·02 (0·95–1·10)	0·59	1·07 (1·00–1·15)	0·045	1·05 (0·99–1·12)	0·12
Number of medical doctors or doctors of osteopathic medicine per 1000 people	1·02 (0·95–1·10)	0·55	1·07 (1·00–1·15)	0·045	1·05 (0·99–1·12)	0·12
Hospital present in ZCTA (reference no hospital present)	1·08 (0·93–1·25)	0·32	1·08 (0·92–1·27)	0·37	1·12 (0·96–1·3)	0·15
Mortality per 1000 people	1·21 (1·14–1·28)	<0·0001	1·17 (1·09–1·24)	<0·0001	1·18 (1·12–1·25)	<0·0001

All models adjusted for time (years since Jan 1, 2016) and included ZCTA population size as an offset. Estimated RR represents exponentiated regression coefficients. The RRs are interpreted as the relative change in ZCTA-level hospitalisation risk for an SD increase in the predictor variable, with the exception of hospital presence in ZCTA, which is not a continuous variable. N_Hosp_ and N_ZCTA_ were the same for all variables except: unemployment rate (opioid N_Hosp_=56 946, N_ZCTA_=374; combined N_Hosp_=103 390, N_ZCTA_=374), social vulnerability index (opioid N_Hosp_=56 393, N_ZCTA_=360; HCV N_Hosp_=46 081, N_ZCTA_=340; combined N_Hosp_=102 474, N_ZCTA_=360), median income (opioid N_Hosp_=56 898, N_ZCTA_=369; HCV N_Hosp_=46 367, N_ZCTA_=347; combined N_Hosp_=103 265, N_ZCTA_=369), and mortality (opioid N_Hosp_=56 801, N_ZCTA_=359; HCV N_Hosp_=46 293, N_ZCTA_=341; combined N_Hosp_=103 094, N_ZCTA_=359). HCV=hepatitis C virus. N_Hosp_=number of hospitalisations. N_ZCTA_=number of ZCTAs. RR=relative risk. ZCTA=ZIP code tabulation area.

* Variable adjusted for in all models.

**Table 3:** Median percent agreement accuracy of predicted opioid-related, HCV-related, and combined hospitalisations across ZCTAs using fully adjusted negative binomial models and previous hospitalisation counts to predict hospitalisation counts in 2021

	Training data	Validation data	*A*_*OP*_	*A*_*HCV*_	*A*_*Comb*_
**Negative binomial model**					
Model scenario 1	2016–20	2021 annual	80·4% (66·8–91·1)	75·2% (61·2–87·7)	84·2% (71·5–92·0)
Model scenario 2	2016–19	2021 annual	80·7% (67·5–90·8)	72·2% (59·2–85·6)	82·1% (67·2–90·9)
Model scenario 3	2016–20	2021 quarter 1	67·4% (42·4–84·7)	67·2% (39·9–82·3)	77·5% (55·8–88·8)
Model scenario 4	2016–20	2021 quarter 2	68·8% (47·7–85·3)	64·9% (39·7–83·3)	75·5% (55·7–87·5)
Model scenario 5	2016–20	2021 quarter 3	70·9% (45·3–86·8)	63·4% (26·0–81·3)	75·7% (52·5–86·8)
Model scenario 6	2016–20	2021 quarter 4	67·2% (45·5–83·4)	60·5% (18·8–78·8)	71·8% (49·9–87·0)
Model scenario 7	2016–19	2021 quarter 1	66·6% (42·2–85·0)	68·9% (37·2–85·4)	77·8% (53·4–87·7)
Model scenario 8	2016–19	2021 quarter 2	68·0% (47·0–85·5)	64·8% (42·1–83·7)	75·4% (55·0–86·7)
Model scenario 9	2016–19	2021 quarter 3	69·6% (44·0–86·8)	64·0% (27·8–82·1)	74·3% (50·2–86·5)
Model scenario 10	2016–19	2021 quarter 4	66·2% (44·8–83·5)	60·6% (18·2–81·3)	70·4% (48·0–86·0)
**Previous hospitalisations**					
Model scenario 1	2020	2021 annual	75·0% (50·0–89·5)	70·3% (50·0–86·9)	80·8% (60·0–92·1)
Model scenario 2	2019	2021 annual	74·3% (50·0–89·5)	66·7% (50·0–83·8)	75·6% (55·3–89·2)
Model scenario 3	2020	2021 quarter 1	66·7% (40·0–84·2)	63·6% (32·3–81·8)	72·7% (50·0–87·0)
Model scenario 4	2020	2021 quarter 2	66·7% (42·1–84·3)	62·5% (30·0–81·0)	71·5% (50·0–85·7)
Model scenario 5	2020	2021 quarter 3	66·7% (37·5–84·8)	57·1% (18·6–80·0)	69·4% (44·4–85·6)
Model scenario 6	2020	2021 quarter 4	66·7% (35·5–80·0)	55·6% (0·0–75·5)	66·7% (40·0–85·7)
Model scenario 7	2019	2021 quarter 1	58·3% (35·4–80·0)	58·7% (26·6–80·0)	69·6% (50·0–85·0)
Model scenario 8	2019	2021 quarter 2	61·5% (40·0–84·1)	58·3% (25·0–80·0)	66·7% (47·5–86·4)
Model scenario 9	2019	2021 quarter 3	57·3% (39·4–80·5)	50·0% (16·0–77·1)	64·1% (44·7–84·1)
Model scenario 10	2019	2021 quarter 4	60·0% (33·3–81·2)	50·0% (0·0–75·0)	62·5% (42·1–81·2)

*A*_*OP*_=agreement accuracy for opioid-related hospitalisations. *A*_*HCV*_=agreement accuracy for HCV-related hospitalisations. *A*_*Comb*_=agreement accuracy for combined hospitalisations. HCV=hepatitis C virus. ZCTA=ZIP code tabulation area.

## Data Availability

Community-level data is publicly available and can be accessed through the Centers for Disease Control and Prevention Social Vulnerability Index and the United States Census Bureau American Community Survey. Patient-level hospitalisation data can be provided by the South Carolina Revenue and Fiscal Affairs Office through an approved data request application.

## Research in context

### Evidence before this study

We searched the literature to identify studies aimed at predicting opioid and hepatitis C virus (HCV) health outcomes at the ZIP code or census tract level. We searched PubMed with no language restrictions for articles published from database inception to May 25, 2023, using the following search terms: ((Opioid[Title]) OR (Overdose[Title]) AND (Hepatitis C Virus[Title]) OR (HCV[Title])) AND ((Zip code[Text Word]) OR (ZCTA[Text Word]) OR (census tract[Text Word]) OR (census block[Text Word])). This search yielded two studies. One study used regression analysis to identify demographic and socioeconomic factors associated with HCV (or HIV) cases and hospitalisations at the ZIP code level but did not implement predictive modelling or incorporate health-care access barriers to identify, predict, or prioritise high-risk communities for targeted interventions. The other study implemented hotspot analyses that were restricted to individuals participating in a substance use treatment programme and were not representative of the general population at risk for HCV or opioid misuse.

### Added value of this study

We identified demographic, socioeconomic, and health-care access variables associated with opioid-related and HCV-related hospitalisations in South Carolina, USA, between 2016 and 2021, and used these variables to predict opioid-related and HCV-related hospitalisations at the ZIP code tabulation area (ZCTA) level using generalised mixed-effects models. Higher proportions of uninsured people, lower income, greater social vulnerability index, rural areas, and poverty were associated with increased opioid-related and HCV-related hospitalisations.

We found that health-care infrastructure (ie, hospital presence and medical personnel) was associated with increased hospitalisation rates, but we did not find evidence that this confounded the association between worse socioeconomic characteristics and hospitalisation risk. Our models showed strong prediction accuracy for opioid-related and HCV-related hospitalisations at the ZCTA level. Prediction accuracy was sustained even when accounting for a 1-year lag in data reporting, indicating the usefulness of this approach for directing field-level interventions in real time. We developed a decision-making framework to identify and prioritise high-risk communities for delivery of mobile health clinics through prioritising hospitalisation risk, sociostructural barriers, and availability of health-care facilities.

### Implications of all the available evidence:

Our findings have important implications for public health response, as they allow for focused interventions in granular geographical regions that consider both community disease risk and availability of other health services. This means public health interventions could be prioritised to high-risk communities of greatest need. Our study serves as a framework that could immediately assist with policies and the distribution of resources through targeted interventions for opioid misuse and HCV. For example, it can guide the decision-making processes for the delivery of mobile health clinics or other field-level interventions to high-risk and medically underserved communities. This approach can easily be adapted for use in other states or for different diseases, serving as a framework for risk assessments, formulating policies, and implementing targeted community-level interventions.
