# Supplementary material for: Identification of factors associated with opioid-related and hepatitis C virus-related hospitalisations at the ZIP code area level in the USA: an ecological and modelling study
Source: Lancet Public Health. Author manuscript; Available in PMC 2024 Jun 10. (PMC11163979; doi:10.1016/S2468-2667(24)00076-8)
Supplement: 1 [file NIHMS1998436-supplement-1.pdf]

THE LANCET
Public Health

Supplementary appendix

This appendix formed part of the original submission and has been peer reviewed. We post it as supplied by the authors.

Supplement to: Gezer F, Howard KA, Litwin AH, Martin NK, Rennert L. Identification of factors associated with opioid-related and hepatitis C virus-related hospitalisations at the ZIP code area level in the USA: an ecological and modelling study. *Lancet Public Health* 2024; **9:** e354–64.

Contents

1. Diagnosis Codes for Hospitalizations 1
2. Prediction Accuracy Metrics 3
3. Model Settings: 4
   3.1 Generalized Linear Mixed Effects Models: 4
   3.2 Conditionally Autoregressive (CAR) Model: 4
   3.3 The Interrupted Time Series (ITS) Model: 5
4. Yearly Descriptive Statistics 6
5. Sensitivity Analysis for Hospital Presence and Size 8
6. Multicollinearity Assessment 10
7. Model Results 12
8. Results for Interrupted Times Series 21
9. Characteristics of High-Risk ZCTAs 26
10. County Level Risk Assessment 29
11. Code 33
References 35

1. **Diagnosis Codes for Hospitalizations**

Table A1: ICD-10 Codes for all inpatient, emergency department, ambulatory, and home health encounters related to opioids and HCV.

OPIOID USE ICD-10 CODES

- F11 – Opioid related disorders
    - F11.1 – Opioid abuse
        - F11.10 – Opioid abuse, uncomplicated
        - F11.11 – Opioid abuse, in remission
        - F11.12 – Opioid abuse with intoxication
            - F11.120 – Opioid abuse with intoxication, uncomplicated
            - F11.121 – Opioid abuse with intoxication delirium
            - F11.122 – Opioid abuse with intoxication with perceptual disturbance
            - F11.129 – Opioid abuse with intoxication, unspecified
        - F11.13 – Opioid abuse with withdrawal
        - F11.14 – Opioid abuse with opioid-induced mood disorder
        - F11.15 – Opioid abuse with opioid-induced psychotic disorder
            - F11.150 – Opioid abuse with opioid-induced psychotic disorder with delusions
            - F11.151 – Opioid abuse with opioid-induced psychotic disorder with hallucinations
            - F11.159 – Opioid abuse with opioid-induced psychotic disorder, unspecified
        - F11.18 – Opioid abuse with other opioid-induced disorder
            - F11.181 – Opioid abuse with opioid-induced sexual dysfunction
            - F11.182 – Opioid abuse with opioid-induced sleep disorder
            - F11.188 – Opioid abuse with other opioid-induced disorder
        - F11.19 – Opioid abuse with unspecified opioid-induced disorder
    - F11.2 – Opioid dependence
        - F11.20 – Opioid dependence, uncomplicated
        - F11.21 – Opioid dependence, in remission
        - F11.22 – Opioid dependence with intoxication
            - F11.220 – Opioid dependence with intoxication, uncomplicated
            - F11.221 – Opioid dependence with intoxication delirium
            - F11.222 – Opioid dependence with intoxication with perceptual disturbance
            - F11.229 – Opioid dependence with intoxication, unspecified
        - F11.23 – Opioid dependence with withdrawal
        - F11.24 – Opioid dependence with opioid-induced mood disorder
        - F11.25 – Opioid dependence with opioid-induced psychotic disorder
            - F11.250 – Opioid dependence with opioid-induced psychotic disorder with delusions
            - F11.251 – Opioid dependence with opioid-induced psychotic disorder with hallucinations
            - F11.259 – Opioid dependence with opioid-induced psychotic disorder, unspecified
        - F11.28 – Opioid dependence with other opioid-induced disorder
            - F11.281 – Opioid dependence with opioid-induced sexual dysfunction
            - F11.282 – Opioid dependence with opioid-induced sleep disorder
            - F11.288 – Opioid dependence with other opioid-induced disorder
        - F11.29 – Opioid dependence with unspecified opioid-induced disorder
    - F11.9 – Opioid use, unspecified
        - F11.90 – Opioid use, unspecified, uncomplicated
        - F11.91 – Opioid use, unspecified, in remission
        - F11.92 – Opioid use, unspecified with intoxication
            - F11.920 – Opioid use, unspecified with intoxication, uncomplicated
            - F11.921 – Opioid use, unspecified with intoxication delirium
            - F11.922 – Opioid use, unspecified with intoxication with perceptual disturbance

        - F11.929 – Opioid use, unspecified with intoxication, unspecified
    - F11.93 – Opioid use, unspecified with withdrawal
    - F11.94 – Opioid use, unspecified with opioid-induced mood disorder
    - F11.95 – Opioid use, unspecified with opioid-induced psychotic disorder
        - F11.950 – Opioid use, unspecified with opioid-induced psychotic disorder with delusions
        - F11.951 – Opioid use, unspecified with opioid-induced psychotic disorder with hallucinations
        - F11.959 – Opioid use, unspecified with opioid-induced psychotic disorder, unspecified
    - F11.98 – Opioid use, unspecified with other specified opioid-induced disorder
        - F11.981 – Opioid use, unspecified with opioid-induced sexual dysfunction
        - F11.982 – Opioid use, unspecified with opioid-induced sleep disorder
        - F11.988 – Opioid use, unspecified with other opioid-induced disorder
    - F11.99 – Opioid use, unspecified with unspecified opioid-induced disorder

HCV ICD-10 CODES

- B17.1 – Acute hepatitis C
    - B17.10 – Acute hepatitis C without hepatic coma
    - B17.11 – Acute hepatitis C with hepatic coma
- B18.2 – Chronic viral hepatitis C
- B19.2 – Unspecified viral hepatitis C
    - B19.20 – Unspecified viral hepatitis C without hepatic coma
    - B19.21 – Unspecified viral hepatitis C with hepatic coma
- Z22.52 – Carrier of viral hepatitis C

2. Prediction Accuracy Metrics

Letting O_i and P_i for i = 1, …, N denote observed and predicted hospitalizations based on a certain model setting for ZCTA i, we calculate the following metrics for model performance comparisons.

Median Agreement Percentage (MAP)

$$\textbf{MAP} = \text{median}\left(\frac{\min(O_i, P_i)}{\max(O_i, P_i)}\right)$$

Median Absolute Error (MAE)

$$\textbf{MAE} = \text{median}(|O_i - P_i|)$$

Root mean Squared Error (RMSE)

$$\textbf{RMSE} = \sqrt{\frac{\sum_{i=1}^{N}(O_i - P_i)^2}{N}}$$

Ranking Agreement Percentage (RAP)

RAP: Overlap percentage between the top 20 ZCTAs based on O_i and P_i

3. Model Settings:

We defined the following stochastic models: generalized linear mixed effects models, Conditionally Autoregressive (CAR) models and the interrupted times series (ITS) models, Sections 1.1, 1.2, and 1.3, respectively.

3.1 Generalized Linear Mixed Effects Models:

Model: $\log\left(E\left[Y_{ij} \mid T_{ij}, X_{ij}\right]\right) = \beta_0 + \beta_1 \times T_{ij} + \gamma \times X_i + b_{i1} + b_{i2} \times T_{ij}$

The outcome variable Y_{ij} is the number of hospitalizations recorded at i-th ZCTA (i = 1, …, 424) for j-th year (j = 2016, 2017, …, 2021). In the quarterly hospitalization counts models the time component j refers to quarters (j = 2016-Q1, 2016-Q2, …, 2021-Q4). The outcomes are the opioid, HCV and combined (opioid and HCV) hospitalizations in the i-th ZCTA at time t. The vector T_{ij} denotes year j for the i-th ZCTA. The variables at the ZCTA level are denoted by X_i (defined in Section 2.2 in the manuscript). Normally distributed random effects b_{i1} and b_{i2} are included in the model for ZCTA and time, respectively. All variables were standardized to a mean of 0 and a standard deviation of 1 for a meaningful comparison of effect sizes. Generalized linear mixed effects models are fitted using Negative Binomial and Poisson family of outcomes.

3.2 Conditionally Autoregressive (CAR) Model:

We adopted the following Bayesian hierarchical spatiotemporal model (1)

Model 1: $\log(\mu_{it}) = \beta_i + S(t) + \eta_t, \eta_t \sim AR(1)$

Model 2: $\log(\mu_{it}) = \beta_i + S(t) + \omega_{it}, \omega_{it} \sim GMRF(\tau\Sigma_{AR(1)} \otimes \Sigma_I)$

Model 3: $\log(\mu_{it}) = \beta + S(t) + \omega_{it}, \omega_{it} \sim GMRF(\tau\Sigma_{AR(1)} \otimes \Sigma_{CAR})$.

In the models, μ_{it} is the hospitalization rate assuming $Y_{it}|\mu_{it} \sim Pois(N_{it}\mu_{it})$ where N_{it} is the population size of the ZCTA as an offset, and i and t are the same as generalized linear mixed effects models above. In all models, temporal trend is captured by $S(t)$. Model 1 included a ZCTA level spatial effect β_i and a first-order autoregressive latent effect η_t. In Model 2 and Model 3, β is the overall intercept and ω_{it} is the spatiotemporal interaction term and followed a Gaussian Markov Random Field (GMRF) with mean 0 and covariance matrix $(\tau\Sigma_T \otimes \Sigma_S)$ that is a Kronecker product of temporal and spatial structures, respectively, and τ is the precision parameter [2]. Model 2 uses an identity matrix for the spatial structure, and Model 3 assumes a CAR model [3] for the spatial structure as

neighbor ZCTAs being similar compared to distant ZCTAs. We added the ZCTA level variables X_i to the above models for the partially and fully adjusted models.

3.3 The Interrupted Time Series (ITS) Model:

$log(E[Y_{it}|T_{it}^*, I_{it}, \mathbf{Z}_{it}; b_i]) = \beta_0 + \beta_1 \times T + \beta_2 \times I_{it} + \beta_3 \times I_{it} \times (T_{it} - t^*)_+ + \boldsymbol{\gamma}'\mathbf{Z_{it}} + b_{i1} + b_{i2} \times T_{ij}$ where Y_{it} is the number of hospitalizations for ZCTA i at time t and assumes a negative Binomial distribution as described in Section A1.1, where $t^*$is the time of the Covid-19 pandemic onset in South Carolina (2020-Q2). Here I_{it}=1 if ZCTA i is in the post-period at time t (i.e., if time $t \geq 2020\ Q2$), and 0 otherwise, and $x_+ = x$ if $x > 0$, and 0 otherwise. In these models, the unit of time is quarter (e.g., time of Covid-19 pandemic onset in SC is quarter 18). Here $\mathbf{Z}_{it}$ is the covariate vector in the model for ZCTA i = 1, …, N at time t (demographic, socio-economic, and healthcare related variables), and $b_i \sim N(0, \sigma_b^2)$ is the random effect for ZCTA.

4. **Yearly Descriptive Statistics**

Table A2: Yearly descriptive characteristics for individuals hospitalized for opioids between 2016 and 2021.

Characteristic	**2016** (N = 9,557)	**2017** (N = 9,304)	**2018** (N = 9,275)	**2019** (N = 9,339)	**2020** (N = 8,730)	**2021** (N = 9,105)
**Age**, Median (IQR)	40 (30 - 54)	41 (30 - 55)	40 (30 - 55)	41 (31 - 56)	40 (31 - 55)	41 (32 - 56)
**Age Group**, N (%)						
18-29 Years	2,325 (24.3)	2,069 (22.2)	2,094 (22.6)	1,913 (20.5)	1,766 (20.2)	1,678 (18.4)
30-44 Years	3,158 (33.0)	3,177 (34.1)	3,214 (34.7)	3,280 (35.1)	3,335 (38.2)	3,495 (38.4)
45-64 Years	3,236 (33.9)	3,154 (33.9)	2,980 (32.1)	3,053 (32.7)	2,665 (30.5)	2,876 (31.6)
65+ Years	838 (8.8)	904 (9.7)	987 (10.6)	1,093 (11.7)	964 (11.0)	1,056 (11.6)
**Sex**, N (%)						
Female	4,870 (51.0)	4,682 (50.3)	4,678 (50.4)	4,664 (49.9)	4,146 (47.5)	4,251 (46.7)
Male	4,687 (49.0)	4,622 (49.7)	4,597 (49.6)	4,675 (50.1)	4,584 (52.5)	4,854 (53.3)
**Race/Ethnicity**, N (%)						
White	8,086 (84.6)	7,850 (84.4)	7,748 (83.5)	7,652 (81.9)	7,210 (82.6)	7,469 (82.0)
Black	1,280 (13.4)	1,259 (13.5)	1,315 (14.2)	1,430 (15.3)	1,250 (14.3)	1,355 (14.9)
Hispanic	48 (0.5)	55 (0.6)	56 (0.6)	67 (0.7)	62 (0.7)	67 (0.7)
Other	143 (1.5)	140 (1.5)	156 (1.7)	190 (2.0)	208 (2.4)	214 (2.4)
**Insurance**, N (%)						
Medicaid	2,345 (24.5)	2,169 (23.3)	2,198 (23.7)	2,069 (22.2)	1,825 (20.9)	2,088 (22.9)
Medicare	2,515 (26.3)	2,422 (26.0)	2,379 (25.6)	2,468 (26.4)	2,102 (24.1)	2,181 (24.0)
Private	2,150 (22.5)	1,997 (21.5)	1,937 (20.9)	1,876 (20.1)	1,678 (19.2)	1,998 (21.9)
Self-Pay/Other	2,547 (26.7)	2,716 (29.2)	2,761 (29.8)	2,926 (31.3)	3,125 (35.8)	2,838 (31.2)

Table A3: Yearly descriptive characteristics for individuals hospitalized for HCV between 2016 and 2021.

Characteristic	**2016** (N = 7,914)	**2017** (N = 7,859)	**2018** (N = 7,869)	**2019** (N = 7,688)	**2020** (N = 7,004)	**2021** (N = 6,199)
**Age**, Median (IQR)	56 (48 - 62)	57 (48 - 62)	57 (47 - 63)	57 (46 - 64)	58 (44 - 64)	58 (44 - 65)
**Age Group**, N (%)						
18-29 Years	473 (6.0)	510 (6.5)	477 (6.1)	475 (6.2)	449 (6.4)	346 (5.6)
30-44 Years	1,082 (13.7)	1,132 (14.4)	1,259 (16.0)	1,315 (17.1)	1,358 (19.4)	1,229 (19.8)
45-64 Years	5,199 (65.7)	4,926 (62.7)	4,617 (58.7)	4,242 (55.2)	3,625 (51.8)	3,047 (49.2)
65+ Years	1,160 (14.7)	1,291 (16.4)	1,516 (19.3)	1,656 (21.5)	1,572 (22.4)	1,577 (25.4)
**Sex**, N (%)						
Female	3,164 (40.0)	3,157 (40.2)	3,237 (41.1)	3,122 (40.6)	2,811 (40.1)	2,437 (39.3)
Male	4,750 (60.0)	4,702 (59.8)	4,631 (58.9)	4,566 (59.4)	4,193 (59.9)	3,762 (60.7)
**Race/Ethnicity**, N (%)						
White	5,240 (66.2)	5,162 (65.7)	5,188 (65.9)	5,222 (67.9)	4,786 (68.3)	4,288 (69.2)
Black	2,470 (31.2)	2,501 (31.8)	2,504 (31.8)	2,278 (29.6)	2,030 (29.0)	1,715 (27.7)
Hispanic	67 (0.8)	56 (0.7)	53 (0.7)	47 (0.6)	50 (0.7)	49 (0.8)
Other	137 (1.7)	140 (1.8)	124 (1.6)	141 (1.8)	138 (2.0)	147 (2.4)
**Insurance**, N (%)						
Medicaid	1,899 (24.0)	1,958 (24.9)	1,908 (24.2)	1,843 (24.0)	1,667 (23.8)	1,465 (23.6)
Medicare	2,826 (35.7)	2,714 (34.5)	2,843 (36.1)	2,794 (36.3)	2,464 (35.2)	2,267 (36.6)
Private	1,972 (24.9)	1,868 (23.8)	1,698 (21.6)	1,587 (20.6)	1,383 (19.7)	1,337 (21.6)
Self-Pay/Other	1,217 (15.4)	1,319 (16.8)	1,420 (18.0)	1,464 (19.0)	1,490 (21.3)	1,130 (18.2)

5. Sensitivity Analysis for Hospital Presence and Size

Table A4: Negative Binomial generalized linear mixed effects model results for opioid, HCV, and combined hospitalizations adjusted for time, hospital presence, and hospital size where the population is an offset.

Variable	**Opioid Hospitalizations ($N_{Hosp.}$ = 56,951, N_{ZCTA} = 376)**			**HCV Hospitalizations ($N_{Hosp.}$ = 46,444, N_{ZCTA} = 353)**			**Combined Hospitalizations ($N_{Hosp.}$ = 103,395, N_{ZCTA} = 376)**		
	**RR**	**CI**	**p-value**	**RR**	**CI**	**p-value**	**RR**	**CI**	**p-value**
**Age** (Ref: % Age 18-29)									
% Age 30-44	1.01	0.93 - 1.1	0.76	0.91	0.84 - 0.98	**0.015**	0.97	0.90 - 1.05	0.44
% Age 45-64	1.13	1.04 - 1.23	**0.0026**	1.12	1.04 - 1.21	**0.0045**	1.14	1.05 - 1.23	**0.0009**
% Age above 65	1.04	0.96 - 1.13	0.32	0.87	0.80 - 0.93	**0.0002**	0.98	0.90 - 1.06	0.55
**Sex** (Ref: % Female)									
% Male	0.98	0.91 - 1.04	0.48	1.01	0.93 - 1.09	0.87	0.97	0.91 - 1.03	0.33
**Race** (Ref: % White)									
% Black	0.97	0.91 - 1.03	0.33	0.98	0.91 - 1.04	0.48	0.96	0.91 - 1.02	0.23
% Other race	0.96	0.90 - 1.02	0.17	1.00	0.93 - 1.07	1	0.97	0.91 - 1.03	0.26
**Ethnicity** (Ref: % Non-Hispanic)									
% Hispanic	0.99	0.93 - 1.05	0.72	0.99	0.93 - 1.06	0.83	0.99	0.93 - 1.06	0.85
**Other**									
Time (year)#	0.99	0.98 - 1.00	**0.016**	0.95	0.93 - 0.98	**<0.0001**	0.97	0.96 - 0.98	**<0.0001**
LH	1.10	1.06 - 1.13	**<0.0001**	1.01	0.99 - 1.03	0.21	1.11	1.07 - 1.16	**<0.0001**
CLOH	-	-	**-**	1.00	1.00 - 1.00	.095	-	-	**-**
**Socioeconomic**									
SVI	1.18	1.12 - 1.25	**<0.0001**	1.16	1.09 - 1.24	**<0.0001**	1.17	1.11 - 1.24	**<0.0001**
Median Income	0.78	0.74 - 0.82	**<0.0001**	0.81	0.76 - 0.86	**<0.0001**	0.79	0.75 - 0.83	**<0.0001**
% in Poverty	1.23	1.16 - 1.31	**<0.0001**	1.22	1.14 - 1.30	**<0.0001**	1.24	1.17 - 1.31	**<0.0001**
% Unemployed	0.91	0.85 - 0.97	**0.0035**	0.93	0.87 – 1.00	**0.035**	0.87	0.82 - 0.93	**<0.0001**
% Labor Force Participation	1.12	1.05 - 1.20	**0.0005**	0.93	0.86 - 0.99	**0.035**	1.03	0.96 - 1.09	0.45
% Rural	1.14	1.07 - 1.22	**<0.0001**	1.06	0.99 - 1.14	0.11	1.1	1.03 - 1.17	**0.0036**
**Healthcare access**									
% Uninsured	1.24	1.17 - 1.31	**<0.0001**	1.18	1.11 - 1.26	**<0.0001**	1.23	1.17 - 1.31	**<0.0001**
PCP Rate per 1000	1.02	0.95 - 1.10	0.57	1.08	1.00 - 1.15	**0.038**	1.05	0.99 - 1.12	0.11
MD/DO Rate per 1000	1.02	0.95 - 1.10	0.53	1.08	1.00 - 1.15	**0.037**	1.05	0.99 - 1.12	0.1
Hospital Present in ZCTA#	1.06	0.88 - 1.27	0.55	2.66	1.81 - 3.89	**<0.0001**	3.46	2.29 - 5.21	**<0.0001**
Hospital Size (Staffed Beds)#	1.01	0.95 - 1.09	0.71	1.27	1.09 - 1.47	**0.0021**	1.20	1.03 - 1.41	**0.022**
**Mortality Rate per 1000**	1.21	1.14 - 1.28	**<0.0001**	1.17	1.09 - 1.24	**<0.0001**	1.18	1.12 - 1.25	**<0.0001**

Estimated relative risk (RR) represents exponentiated regression coefficients. Associated 95% confidence intervals (CI) and p-values for each RR are provided. $N_{Hosp.}$ and N_{ZCTA} denotes the number of hospitalizations and ZCTAs included in the analysis for all variables, with the exception of: unemployment rate (opioid: $N_{Hosp.}$ = 56,946, N_{ZCTA} = 374; combined: $N_{Hosp.}$ = 103,390, N_{ZCTA} = 374), SVI (opioid: $N_{Hosp.}$ = 56,393, N_{ZCTA} = 360; HCV: $N_{Hosp.}$ = 46,081, N_{ZCTA} = 340; combined: $N_{Hosp.}$ = 102,474, N_{ZCTA} = 360), median income (opioid: $N_{Hosp.}$ = 56,898, N_{ZCTA} = 369; HCV: $N_{Hosp.}$ = 46,367, N_{ZCTA} = 347; combined: $N_{Hosp.}$ = 103,265, N_{ZCTA} = 369), and mortality rate (opioid: $N_{Hosp.}$ = 56,801, N_{ZCTA} = 359; HCV: $N_{Hosp.}$ = 46,293, N_{ZCTA} = 341; combined: $N_{Hosp.}$ = 103,094, N_{ZCTA} = 359). The RR are interpreted as the relative change in ZCTA-level hospitalization risk for a standard deviation increase in the predictor variable. HCV: hepatitis C virus, SVI: social vulnerability index, PCP: primary care physicians MD/DO: medical doctors or doctor of osteopathic medicine, LH: Lagged hospitalization counts, CLOH: Cumulative lagged opioid hospitalization counts, and Ref indicates the reference group. **Bolded p-values** represent statistical significance ($P<0.05$).

[#] Variable adjusted for in all models.

6. Multicollinearity Assessment

We investigated the multicollinearity of variables by calculating the variance inflation factor (VIF) for each variable based on the Negative Binomial and Poisson models for each disease outcome. The VIF determines the magnitude of multicollinearity by measuring how much the variance of the estimated regression coefficients is inflated due to multicollinearity. Commonly, VIF greater than 10 is considered that the variable is causing multicollinearity and to be removed from the model [4]. Our initial fully adjusted model showed that PCP Rate and MD/DO Rate had very high VIF measures, hence we fitted separate models that include PCP Rate and MD/DO Rate separately. **Table A19** shows the VIF values for all variables when PCP Rate and MD/DO Rate were not included in the models at the same time. The VIF values for PCP Rate and MD/DO Rate are obtained from the separate models. **Table A19** shows that VIF values for all variables are under the threshold of 10.

Table A5: Variance inflation factor (VIF) calculated for each variable. VIF for PCP and MD/DO rates were obtained from separate models that include either of these variables.

	Negative Binomial Models		
**Variable**	**Opioid**	**HCV**	**Comb.**
% Age 30-44	1.95	1.94	1.92
% Age 45-64	1.61	1.63	1.53
% Age above 65	2.88	2.71	2.59
% Male	1.13	1.13	1.13
% Black	1.82	1.81	1.80
% Other race	2.18	1.98	2.15
% Hispanic	2.59	2.49	2.56
Time	1.00	1.04	1.01
Lag Counts	1.32	1.09	1.48
SVI	2.37	2.35	2.28
% Uninsured	1.81	1.79	1.79
Median Income	3.93	4.01	4.02
% in Poverty	2.61	2.79	2.67
% Unemployed	1.19	1.18	1.17
% Labor Force Participation	2.32	2.35	2.27
% Rural	2.67	2.42	2.72
PCP Rate*	214.0	296.4	261.7
MD/DO Rate*	212.2	295.1	259.9
PCP Rate	1.08	1.08	1.09
MD/DO Rate	1.07	1.07	1.08
Hospital Present	1.29	1.26	1.29
Mortality Rate	2.39	2.17	2.29

HCV: hepatitis C virus, SVI: social vulnerability index, PCP: primary care physicians MD/DO: medical doctors or doctor of osteopathic medicine. * VIF for PCP and MD/DO rates when both variables are included in the same model.

7. **Model Results**

Table A6: Fully adjusted Negative Binomial generalized linear mixed effects model results for opioid, HCV, and combined hospitalizations.

Variable	Opioid Hospitalizations			HCV Hospitalizations			Combined Hospitalizations		
	**RR**	**CI**	**p-value**	**RR**	**CI**	**p-value**	**RR**	**CI**	**p-value**
**Age** (Ref: % Age 18-29)									
% Age 30-44	1.12	1.04 - 1.20	**0.0013**	1.01	0.94 - 1.09	0.76	1.05	0.98 - 1.12	0.15
% Age 45-64	1.11	1.03 - 1.21	**0.0069**	1.04	0.96 - 1.13	0.31	1.09	1.02 - 1.18	**0.018**
% Age above 65	1.03	0.94 - 1.12	0.57	0.91	0.84 – 1.00	**0.045**	0.96	0.88 - 1.04	0.3
**Sex** (Ref: % Female)									
% Male	1.00	0.94 - 1.06	0.95	1.07	0.99 - 1.15	0.088	1.03	0.98 - 1.09	0.27
**Race** (Ref: % White)									
% Black	0.82	0.77 - 0.87	**<0.0001**	0.86	0.81 - 0.92	**<0.0001**	0.85	0.8 - 0.90	**<0.0001**
% Other race	1.03	0.96 - 1.10	0.43	0.97	0.9 - 1.05	0.42	1.01	0.95 - 1.07	0.78
**Ethnicity** (Ref: % Non-Hispanic)									
% Hispanic	0.85	0.79 - 0.92	**0.0001**	0.96	0.88 - 1.04	0.34	0.89	0.83 - 0.96	**0.002**
**Other**									
Time (year)	1.00	0.98 - 1.01	0.54	0.95	0.93 - 0.97	**<0.0001**	0.97	0.96 - 0.99	**0.0001**
LH	1.13	1.09 - 1.17	**<0.0001**	1.02	1.00 - 1.03	0.065	1.15	1.11 - 1.20	**<0.0001**
CLOH	-	-	**-**	1.01	0.99 - 1.03	0.32	-	-	**-**
**Socioeconomic**									
SVI	1.05	0.99 - 1.13	0.12	1.02	0.95 - 1.11	0.57	1.05	0.99 - 1.12	0.13
Median Income	0.81	0.74 - 0.88	**<0.0001**	0.75	0.68 - 0.83	**<0.0001**	0.81	0.74 - 0.88	**<0.0001**
% in Poverty	1.06	0.97 - 1.16	0.23	1.02	0.92 - 1.14	0.67	1.04	0.96 - 1.14	0.34
% Unemployed	0.94	0.89 - 1.00	**0.035**	0.92	0.86 - 0.97	**0.0033**	0.91	0.85 - 0.97	**0.0046**
% Labor Force Participation	1.06	0.98 - 1.15	0.14	0.96	0.87 - 1.05	0.35	1.03	0.96 - 1.12	0.42
% Rural	0.98	0.91 - 1.04	0.48	0.85	0.79 - 0.92	**<0.0001**	0.96	0.9 - 1.02	0.21
**Healthcare access**									
% Uninsured	1.17	1.1 - 1.24	**<0.0001**	1.09	1.02 - 1.17	**0.016**	1.12	1.05 - 1.18	**0.0002**
PCP Rate per 1000	1.16	0.85 - 1.59	0.35	0.94	0.65 - 1.36	0.75	1.08	0.82 - 1.41	0.58
MD/DO Rate per 1000	1.10	0.86 - 1.40	0.45	0.95	0.72 - 1.26	0.73	1.04	0.85 - 1.28	0.69
Hospital Present in ZCTA	1.06	0.95 - 1.19	0.32	1.03	0.90 - 1.18	0.64	1.02	0.92 - 1.14	0.66
**Mortality Rate per 1000**	1.06	0.98 - 1.14	0.17	1.10	1.01 - 1.20	**0.036**	1.09	1.02 - 1.17	**0.017**

Estimated relative risk (RR) represents exponentiated regression coefficients. Associated 95% confidence intervals (CI) and p-values for each RR are provided. The interpretation of the relative risk (RR) is conditional and hence convoluted due to other factors in the model and should not be interpreted as direct effects on the outcome. HCV: hepatitis C virus, SVI: social vulnerability index, PCP: primary care physicians MD/DO: medical doctors or doctor of osteopathic medicine, LH: Lagged hospitalization counts, CLOH: Cumulative lagged opioid hospitalization counts, and Ref indicates the reference group.

Results for random effects

Table A7 shows that univariate models have greater ZCTA random effect variance compared to the fully adjusted models for all hospitalization types and using negative Binomial and Poisson family of generalized linear mixed effects models. On the other hand, the variance for the time random effect was always very small. Moreover, we performed a likelihood ratio test to compare models with and without random effects in terms of their goodness of fit. We test the hypotheses: H_o: The full and reduced models fit the data equally well (this indicates the utility of the reduced model), and H_A: The full model fits the data significantly better than the reduced model (which indicates the usage of the full model). We compared the fully adjusted Negative Binomial model with ZCTA and time random effects to the Model 1: a fully adjusted Negative Binomial model that only includes ZCTA random effect, Model 2: a fully adjusted Negative Binomial model that only includes time random effect, and Model 3: a generalized linear model that does not include any random effects. **Table A8** shows that the main fully adjusted Negative Binomial models had better goodness of fit compared to other reduced model structures.

Table A7: Variances of ZCTA and time as the random effect in the Negative Binomial generalized linear mixed effects models using fully adjusted and univariate models for annual hospitalization data.

	**Opioid Hospitalizations**		**HCV Hospitalizations**		**Combined Hospitalizations**	
	**Fully Adjusted**	**Univariate**	**Fully Adjusted**	**Univariate**	**Fully Adjusted**	**Univariate**
**Negative Binomial Model**						
ZCTA	0.129	0.285	0.179	0.362	0.111	0.319
Time	0.001	0.001	0.002	0.002	0.0003	0.0003

Table A8: Likelihood ratio test comparing models with different random effect structure. Reference model is the fully adjusted Negative Binomial model that includes both ZCTA and time as the random effect. Comparison models are defined as follows: Model 1 is a fully adjusted Negative Binomial model that only includes ZCTA random effect, Model 2 is a fully adjusted Negative Binomial model that only includes time random effect, and Model 3 is a generalized linear model that does not include any random effects.

	**Opioid Hospitalizations**	**HCV Hospitalizations**	**Combined Hospitalizations**
**Negative Binomial Model**			
Model 1	0.005	<0.001	0.009
Model 2	<0.001	<0.001	<0.001
Model 3	<0.001	<0.001	<0.001

Table A9: Poisson generalized linear mixed effects model results for opioid, HCV, and combined hospitalizations adjusted for time where the population is an offset.

Variable	Opioid Hospitalizations			HCV Hospitalizations			Combined Hospitalizations		
	**RR**	**CI**	**p-value**	**RR**	**CI**	**p-value**	**RR**	**CI**	**p-value**
**Age** (Ref: % Age 18-29)									
% Age 30-44	1.01	0.93 - 1.09	0.81	0.89	0.82 - 0.97	**0.0059**	0.96	0.89 - 1.04	0.33
% Age 45-64	1.12	1.03 - 1.21	**0.0056**	1.09	1.01 - 1.18	**0.034**	1.11	1.03 - 1.20	**0.0054**
% Age above 65	1.04	0.96 - 1.13	0.37	0.85	0.79 - 0.92	**<0.0001**	0.97	0.89 - 1.05	0.39
**Sex** (Ref: % Female)									
% Male	0.98	0.92 - 1.04	0.49	0.99	0.91 - 1.08	0.87	0.97	0.91 - 1.03	0.27
**Race** (Ref: % White)									
% Black	0.97	0.91 - 1.03	0.37	0.98	0.92 - 1.05	0.64	0.97	0.91 - 1.03	0.31
% Other race	0.96	0.91 - 1.02	0.22	1.01	0.94 - 1.09	0.79	0.97	0.92 - 1.03	0.39
**Ethnicity** (Ref: % Non-Hispanic)									
% Hispanic	0.99	0.93 - 1.06	0.84	1.01	0.94 - 1.07	0.87	1.00	0.94 - 1.07	0.9
**Other**									
Time (year)#	0.99	0.98 - 1.00	**0.0071**	0.95	0.93 - 0.97	**<0.0001**	0.97	0.96 - 0.98	**<0.0001**
LH	1.08	1.05 - 1.11	**<0.0001**	1.01	0.99 - 1.02	0.27	1.10	1.07 - 1.13	**<0.0001**
CLOH	-	-	**-**	1.01	1.00 - 1.03	**0.039**	-	-	**-**
**Socioeconomic**									
SVI	1.18	1.12 - 1.25	**<0.0001**	1.17	1.09 - 1.24	**<0.0001**	1.17	1.1 - 1.24	**<0.0001**
Median Income	0.78	0.74 - 0.82	**<0.0001**	0.81	0.76 - 0.86	**<0.0001**	0.79	0.75 - 0.84	**<0.0001**
% in Poverty	1.23	1.16 - 1.31	**<0.0001**	1.22	1.15 - 1.31	**<0.0001**	1.24	1.17 - 1.31	**<0.0001**
% Unemployed	0.91	0.85 - 0.97	**0.0036**	0.93	0.87 – 1.00	**0.037**	0.87	0.82 - 0.93	**<0.0001**
% Labor Force Participation	1.12	1.05 - 1.19	**0.0004**	0.91	0.85 - 0.98	**0.014**	1.02	0.95 - 1.08	0.62
% Rural	1.10	1.04 - 1.17	**0.0004**	1.02	0.96 - 1.09	0.54	1.06	0.99 - 1.12	0.073
**Healthcare access**									
% Uninsured	1.24	1.17 - 1.31	**<0.0001**	1.18	1.11 - 1.26	**<0.0001**	1.24	1.17 - 1.31	**<0.0001**
PCP Rate per 1000	1.02	0.95 - 1.09	0.59	1.07	1.00 - 1.15	**0.045**	1.05	0.99 - 1.12	0.12
MD/DO Rate per 1000	1.02	0.95 - 1.10	0.56	1.07	1.00 - 1.15	**0.045**	1.05	0.99 - 1.12	0.12
Hospital Present in ZCTA	1.08	0.93 - 1.25	0.32	1.08	0.92 - 1.27	0.36	1.12	0.96 - 1.30	0.15
**Mortality Rate per 1000**	1.21	1.14 - 1.28	**<0.0001**	1.17	1.09 - 1.24	**<0.0001**	1.18	1.12 - 1.25	**<0.0001**

Estimated relative risk (RR) represents exponentiated regression coefficients. Associated 95% confidence intervals (CI) and p-values for each RR are provided. The RR are interpreted as the relative change in ZCTA-level hospitalization risk for a standard deviation increase in the predictor variable. HCV: hepatitis C virus, SVI: social vulnerability index, PCP: primary care physicians MD/DO: medical doctors or doctor of osteopathic medicine, LH: Lagged hospitalization counts, CLOH: Cumulative lagged opioid hospitalization counts, and Ref indicates the reference group. **Bolded p-values** represent statistical significance (P<0.05).

Variable adjusted for in all models.

Table A10: Fully adjusted Poisson generalized linear mixed effects model results for opioid, HCV, and combined hospitalizations.

Variable	Opioid Hospitalizations			HCV Hospitalizations			Combined Hospitalizations		
	**RR**	**CI**	**p-value**	**RR**	**CI**	**p-value**	**RR**	**CI**	**p-value**
**Age** (Ref: % Age 18-29)									
% Age 30-44	1.12	1.05 - 1.21	**0.0004**	1.01	0.94 - 1.09	0.77	1.05	0.99 - 1.13	0.13
% Age 45-64	1.12	1.04 - 1.22	**0.0047**	1.04	0.96 - 1.13	0.33	1.10	1.02 - 1.19	**0.014**
% Age above 65	1.03	0.94 - 1.13	0.53	0.91	0.84 – 1.00	**0.046**	0.96	0.88 - 1.04	0.31
**Sex** (Ref: % Female)									
% Male	1.00	0.94 - 1.06	0.87	1.07	0.99 - 1.16	0.086	1.03	0.97 - 1.09	0.32
**Race** (Ref: % White)									
% Black	0.82	0.77 - 0.87	**<0.0001**	0.86	0.81 - 0.92	**<0.0001**	0.85	0.8 - 0.89	**<0.0001**
% Other race	1.03	0.96 - 1.1	0.44	0.97	0.90 - 1.05	0.44	1.01	0.95 - 1.07	0.77
**Ethnicity** (Ref: % Non-Hispanic)									
% Hispanic	0.85	0.79 - 0.92	**0.0004**	0.96	0.88 - 1.04	0.35	0.89	0.83 - 0.96	**0.0019**
**Other**									
Time (year)	0.99	0.98 - 1.01	0.43	0.94	0.92 - 0.97	**<0.0001**	0.97	0.96 - 0.99	**0.0004**
LH	1.10	1.07 - 1.14	**<0.0001**	1.01	0.99 - 1.02	0.39	1.12	1.09 - 1.16	**<0.0001**
CLOH	-	-	**-**	1.01	1.00 - 1.03	0.12	-	-	**-**
**Socioeconomic**									
SVI	1.05	0.99 - 1.13	0.13	1.02	0.95 - 1.11	0.57	1.05	0.99 - 1.12	0.13
Median Income	0.8	0.74 - 0.87	**<0.0001**	0.75	0.68 - 0.83	**<0.0001**	0.8	0.74 - 0.87	**<0.0001**
% in Poverty	1.06	0.96 - 1.16	0.25	1.02	0.92 - 1.14	0.66	1.04	0.96 - 1.14	0.34
% Unemployed	0.94	0.89 - 1.00	**0.035**	0.92	0.86 - 0.97	**0.003**	0.91	0.85 - 0.97	**0.0043**
% Labor Force Participation	1.06	0.98 - 1.16	0.13	0.96	0.87 - 1.05	0.33	1.03	0.95 - 1.12	0.42
% Rural	0.96	0.9 - 1.03	0.25	0.85	0.79 - 0.92	**<0.0001**	0.94	0.88 - 1.01	0.071
**Healthcare access**									
% Uninsured	1.17	1.10 - 1.25	**<0.0001**	1.09	1.01 - 1.17	**0.018**	1.12	1.06 - 1.19	**0.0004**
PCP Rate per 1000	1.16	0.85 - 1.59	0.36	0.94	0.65 - 1.36	0.74	1.07	0.82 - 1.41	0.61
MD/DO Rate per 1000	1.10	0.86 - 1.40	0.46	0.95	0.72 - 1.26	0.72	1.04	0.84 - 1.28	0.72
Hospital Present in ZCTA	1.07	0.96 - 1.20	0.23	1.03	0.90 - 1.18	0.65	1.04	0.93 - 1.16	0.50
**Mortality Rate per 1000**	1.05	0.97 - 1.14	0.21	1.10	1.01 - 1.20	**0.037**	1.09	1.01 - 1.17	**0.024**

Estimated relative risk (RR) represents exponentiated regression coefficients. Associated 95% confidence intervals (CI) and p-values for each RR are provided. The interpretation of the relative risk (RR) is conditional and hence convoluted due to other factors in the model and should not be interpreted as direct effects on the outcome. HCV: hepatitis C virus, SVI: social vulnerability index, PCP: primary care physicians MD/DO: medical doctors or doctor of osteopathic medicine, LH: Lagged hospitalization counts, CLOH: Cumulative lagged opioid hospitalization counts, and Ref indicates the reference group.

Table A11: Conditionally Autoregressive (CAR) model results for opioid, HCV, and combined hospitalizations adjusted for time where the population is an offset.

Variable	Opioid Hospitalizations		HCV Hospitalizations		Combined Hospitalizations	
	RR	CI	RR	CI	RR	CI
**Age** (Ref: % Age 18-29)						
% Age 30-44	0.99	0.98 - 1.01	0.96	0.94 - 0.97	0.97	0.95 - 0.99
% Age 45-64	0.99	0.95 - 1.03	0.93	0.90 - 0.97	0.91	0.87 - 0.95
% Age above 65	1.06	1.02 - 1.11	0.97	0.93 - 1.01	1.00	0.96 - 1.05
**Sex** (Ref: % Female)						
% Male	1.03	0.99 - 1.07	1.10	1.06 - 1.15	1.05	1.01 - 1.09
**Race** (Ref: % White)						
% Black	1.00	0.98 - 1.01	0.95	0.94 - 0.97	0.97	0.95 - 0.99
% Other race	1.01	0.97 - 1.04	1.10	1.07 - 1.14	1.02	0.99 - 1.06
**Ethnicity** (Ref: % Non-Hispanic)						
% Hispanic	0.99	0.96 - 1.03	1.06	1.03 - 1.09	1.01	0.98 - 1.05
**Other**						
Time (year)#	0.98	0.97 - 1.00	0.95	0.94 - 0.96	0.97	0.96 - 0.99
LH	1.18	1.15 - 1.21	1.20	1.17 - 1.23	1.18	1.14 - 1.22
CLOH	-	-	1.08	1.05 - 1.11	-	-
**Socioeconomic**						
SVI	1.17	1.14 - 1.21	1.19	1.16 - 1.22	1.19	1.16 - 1.22
Median Income	0.77	0.76 - 0.79	0.77	0.75 - 0.79	0.76	0.74 - 0.78
% in Poverty	1.26	1.21 - 1.30	1.29	1.25 - 1.33	1.31	1.26 - 1.35
% Unemployed	0.99	0.95 - 1.03	1.04	1.00 - 1.07	1.01	0.96 - 1.06
% Labor Force Participation	1.14	1.10 - 1.18	1.02	0.99 - 1.06	1.11	1.07 - 1.15
% Rural	1.11	1.07 - 1.15	1.02	0.98 - 1.05	1.07	1.04 - 1.11
**Healthcare access**						
% Uninsured	1.25	1.22 - 1.28	1.24	1.21 - 1.28	1.26	1.23 - 1.29
PCP Rate per 1000	1.05	0.81 - 1.33	0.84	0.61 - 1.10	0.98	0.78 - 1.21
MD/DO Rate per 1000	1.00	0.78 - 1.23	0.88	0.68 - 1.11	0.97	0.81 - 1.15
Hospital Present in ZCTA	1.03	0.97 - 1.09	1.12	1.06 - 1.19	1.09	1.02 - 1.16
**Mortality Rate per 1000**	1.23	1.19 - 1.27	1.19	1.15 - 1.23	1.24	1.20 - 1.28

Estimated relative risk (RR) represents exponentiated regression coefficients. Associated 95% confidence intervals (CI) are provided. The RR are interpreted as the relative change in ZCTA-level hospitalization risk for a standard deviation increase in the predictor variable. HCV: hepatitis C virus, SVI: social vulnerability index, PCP: primary care physicians MD/DO: medical doctors or doctor of osteopathic medicine, LH: Lagged hospitalization counts, CLOH: Cumulative lagged opioid hospitalization counts, and Ref indicates the reference group.

Variable adjusted for in all models.

Table A12: Fully adjusted Conditionally Autoregressive (CAR) model results for opioid, HCV, and combined hospitalizations.

Variable	Opioid Hospitalizations		HCV Hospitalizations		Combined Hospitalizations	
	RR	CI	RR	CI	RR	CI
**Age** (Ref: % Age 18-29)						
% Age 30-44	1.07	1.03 - 1.10	1.01	0.98 - 1.04	1.01	0.97 - 1.04
% Age 45-64	1.08	1.04 - 1.12	1.02	0.98 - 1.06	1.04	1.00 - 1.07
% Age above 65	1.00	0.95 - 1.04	0.94	0.91 - 0.98	0.94	0.90 - 0.97
**Sex** (Ref: % Female)						
% Male	1.04	1.01 - 1.07	1.12	1.08 - 1.16	1.07	1.04 - 1.10
**Race** (Ref: % White)						
% Black	0.81	0.79 - 0.84	0.90	0.87 - 0.93	0.85	0.82 - 0.87
% Other race	0.96	0.93 - 1.00	0.89	0.85 - 0.93	0.94	0.91 - 0.98
**Ethnicity** (Ref: % Non-Hispanic)						
% Hispanic	0.96	0.92 - 1.00	1.04	1.00 - 1.09	0.98	0.94 - 1.02
**Other**						
Time (year)	0.98	0.97 - 1.00	0.95	0.94 - 0.96	0.97	0.96 - 0.99
LH	1.18	1.15 - 1.21	1.15	1.13 - 1.18	1.18	1.14 - 1.22
CLOH	-	-	0.99	0.97 - 1.02	-	-
**Socioeconomic**						
SVI	1.05	1.02 - 1.08	1.03	1.00 - 1.07	1.05	1.02 - 1.08
Median Income	0.79	0.75 - 0.82	0.79	0.75 - 0.83	0.82	0.79 - 0.85
% in Poverty	1.00	0.96 - 1.05	0.98	0.93 - 1.04	1.03	0.99 - 1.08
% Unemployed	0.98	0.95 - 1.01	0.99	0.96 - 1.02	0.97	0.94 - 1.00
% Labor Force Participation	1.02	0.98 - 1.06	0.95	0.91 - 0.99	1.00	0.97 - 1.05
% Rural	1.04	1.00 - 1.08	0.96	0.92 - 0.99	1.03	0.99 - 1.06
**Healthcare access**						
% Uninsured	1.10	1.06 - 1.13	1.08	1.04 - 1.11	1.08	1.05 - 1.11
PCP Rate per 1000	1.13	0.90 - 1.37	0.91	0.68 - 1.19	1.02	0.86 - 1.22
MD/DO Rate per 1000	1.08	0.92 - 1.26	0.92	0.74 - 1.11	1.00	0.87 - 1.15
Hospital Present in ZCTA	0.97	0.93 - 1.01	0.96	0.92 - 1.01	0.96	0.91 - 1.01
**Mortality Rate per 1000**	1.08	1.04 - 1.12	1.13	1.08 - 1.18	1.10	1.07 - 1.14
ρ_s	0.93	0.87 - 0.98	0.87	0.78 - 0.94	0.67	0.52 - 0.81
ρ_t	0.06	0.00 - 0.16	0.01	0.00 - 0.04	0.03	0.00 - 0.10

Estimated relative risk (RR) represents exponentiated regression coefficients. Associated 95% confidence intervals (CI) are provided. The interpretation of the relative risk (RR) is conditional and hence convoluted due to other factors in the model and should not be interpreted as direct effects on the outcome. HCV: hepatitis C virus, SVI: social vulnerability index, PCP: primary care physicians MD/DO: medical doctors or doctor of osteopathic medicine, LH: Lagged hospitalization counts, CLOH: Cumulative lagged opioid hospitalization counts, and Ref indicates the reference group.

Table A13: Prediction accuracy metrics for all models.

Training Data	Validation Data	MAP			MAE			RMSE			RAP		
		Opioid	HCV	Comb.	Opioid	HCV	Comb.	Opioid	HCV	Comb.	Opioid	HCV	Comb.
**Negative Binomial Regression**													
**Fully Adjusted Model**													
2016-2020	2021-Ann	80.4%	75.2%	84.2%	4.6	5.0	7.3	7.2	7.8	11.9	85.0%	75.0%	80.0%
2016-2019	2021-Ann	80.7%	72.2%	82.1%	4.8	6.4	8.8	7.6	10.0	14.6	90.0%	75.0%	75.0%
2016-2020	2021-Q1	67.4%	67.2%	77.5%	2.0	1.8	2.9	2.9	2.6	4.3	85.0%	70.0%	80.0%
2016-2020	2021-Q2	68.8%	64.9%	75.5%	2.3	2.1	3.4	3.5	3.1	5.2	90.0%	65.0%	85.0%
2016-2020	2021-Q3	70.9%	63.4%	75.7%	2.2	2.3	3.5	3.6	3.5	5.5	75.0%	55.0%	70.0%
2016-2020	2021-Q4	67.2%	60.5%	71.8%	2.1	2.2	3.3	3.1	3.1	5.0	65.0%	60.0%	70.0%
2016-2019	2021-Q1	66.6%	68.9%	77.8%	2.0	1.6	3.1	3.0	2.2	4.7	85.0%	70.0%	80.0%
2016-2019	2021-Q2	68.0%	64.8%	75.4%	2.3	2.0	3.5	3.5	2.8	5.3	90.0%	65.0%	85.0%
2016-2019	2021-Q3	69.6%	64.0%	74.3%	2.3	2.1	3.7	3.7	3.2	5.9	75.0%	55.0%	70.0%
2016-2019	2021-Q4	66.2%	60.6%	70.4%	2.2	2.0	3.8	3.3	2.8	5.8	65.0%	65.0%	70.0%
**Univariate Model**													
2016-2020	2021-Ann	79.5%	74.4%	82.7%	4.6	4.6	4.6	7.2	7.9	11.9	85.0%	75.0%	80.0%
2016-2019	2021-Ann	79.0%	72.2%	80.2%	5.0	5.0	5.0	7.8	10.1	14.7	85.0%	75.0%	75.0%
2016-2020	2021-Q1	65.2%	64.7%	76.9%	2.0	1.8	2.9	2.9	2.6	4.3	85.0%	70.0%	80.0%
2016-2020	2021-Q2	68.3%	63.1%	74.5%	2.3	2.1	3.4	3.5	3.1	5.2	90.0%	65.0%	85.0%
2016-2020	2021-Q3	67.5%	61.9%	73.8%	2.2	2.3	3.5	3.6	3.6	5.6	75.0%	55.0%	70.0%
2016-2020	2021-Q4	64.9%	56.6%	70.0%	2.1	2.3	3.3	3.1	3.1	5.0	65.0%	60.0%	70.0%
2016-2019	2021-Q1	64.6%	66.3%	76.2%	2.0	1.6	3.1	3.0	2.2	4.8	85.0%	70.0%	80.0%
2016-2019	2021-Q2	67.4%	63.8%	74.6%	2.3	2.0	3.5	3.5	2.8	5.3	90.0%	65.0%	85.0%
2016-2019	2021-Q3	66.8%	62.3%	72.3%	2.3	2.1	3.7	3.7	3.2	5.9	80.0%	55.0%	70.0%
2016-2019	2021-Q4	63.3%	57.9%	67.9%	2.2	2.0	3.8	3.3	2.8	5.8	70.0%	60.0%	70.0%
**Poisson Regression**													
**Fully Adjusted Model**													
2016-2020	2021-Ann	80.3%	75.2%	84.0%	4.5	5.0	7.3	7.2	7.8	12.0	85.0%	75.0%	80.0%
2016-2019	2021-Ann	80.5%	72.3%	82.0%	4.9	6.3	8.9	7.6	10.0	14.7	90.0%	75.0%	70.0%
2016-2020	2021-Q1	67.4%	67.1%	77.6%	2.0	2.0	2.0	2.9	2.6	4.3	85.0%	70.0%	80.0%
2016-2020	2021-Q2	68.8%	65.0%	75.4%	1.8	1.8	1.8	3.5	3.1	5.2	90.0%	65.0%	85.0%
2016-2020	2021-Q3	70.8%	63.3%	75.5%	2.9	2.9	2.9	3.6	3.5	5.6	75.0%	55.0%	70.0%
2016-2020	2021-Q4	67.3%	60.4%	71.9%	2.3	2.3	2.3	3.1	3.1	5.0	65.0%	60.0%	70.0%
2016-2019	2021-Q1	66.6%	64.9%	77.7%	2.1	2.1	2.1	3.0	2.9	4.8	85.0%	70.0%	80.0%
2016-2019	2021-Q2	67.9%	62.4%	75.3%	3.4	3.4	3.4	3.5	3.4	5.3	90.0%	65.0%	85.0%
2016-2019	2021-Q3	69.6%	60.8%	74.2%	2.2	2.2	2.2	3.7	3.9	5.9	75.0%	50.0%	70.0%

2016-2019	2021-Q4	66.2%	57.4%	70.2%	2.3	2.3	2.3	3.3	3.7	5.8	65.0%	65.0%	70.0%
**Univariate Model**													
2016-2020	2021-Ann	79.4%	74.4%	82.7%	4.6	5.0	7.3	7.2	7.9	12.0	85.0%	75.0%	80.0%
2016-2019	2021-Ann	78.9%	72.2%	80.2%	5.0	6.4	9.0	7.9	10.0	14.8	90.0%	75.0%	70.0%
2016-2020	2021-Q1	65.2%	64.7%	76.9%	2.0	2.0	2.0	2.9	2.6	4.3	85.0%	70.0%	80.0%
2016-2020	2021-Q2	68.3%	63.1%	74.5%	1.8	1.8	1.8	3.5	3.1	5.2	90.0%	65.0%	85.0%
2016-2020	2021-Q3	67.5%	61.8%	73.7%	2.9	2.9	2.9	3.6	3.6	5.6	75.0%	55.0%	70.0%
2016-2020	2021-Q4	64.9%	56.5%	69.9%	2.3	2.3	2.3	3.1	3.1	5.1	65.0%	60.0%	70.0%
2016-2019	2021-Q1	64.6%	61.8%	76.2%	2.1	2.1	2.1	3.0	3.0	4.8	85.0%	70.0%	80.0%
2016-2019	2021-Q2	67.5%	60.9%	74.6%	3.4	3.4	3.4	3.5	3.4	5.3	90.0%	65.0%	85.0%
2016-2019	2021-Q3	66.8%	59.5%	72.3%	2.2	2.2	2.2	3.7	4.0	5.9	80.0%	50.0%	70.0%
2016-2019	2021-Q4	63.3%	55.3%	67.9%	2.3	2.3	2.3	3.3	3.7	5.8	70.0%	55.0%	70.0%
**Conditionally Autoregressive Model**													
**Fully Adjusted Model**													
2016-2020	2021-Ann	74.8%	70.1%	78.0%	6.8	5.8	10.8	11.4	9.5	16.4	85.0%	75.0%	80.0%
2016-2019	2021-Ann	75.1%	70.7%	77.1%	7.6	7.1	11.7	16.7	11.2	20.1	85.0%	80.0%	70.0%
2016-2020	2021-Q1	62.4%	64.0%	73.2%	2.4	2.2	3.4	3.8	2.9	5.1	75.0%	60.0%	70.0%
2016-2020	2021-Q2	64.3%	60.2%	70.5%	2.6	2.5	4.0	4.0	3.7	6.2	85.0%	55.0%	80.0%
2016-2020	2021-Q3	66.0%	60.9%	73.5%	2.6	2.5	4.0	4.1	3.8	6.6	80.0%	55.0%	70.0%
2016-2020	2021-Q4	66.9%	58.3%	71.7%	2.3	2.3	3.5	3.6	3.6	5.0	65.0%	65.0%	70.0%
2016-2019	2021-Q1	62.7%	63.8%	71.9%	2.4	2.3	3.6	3.8	3.3	5.6	75.0%	60.0%	75.0%
2016-2019	2021-Q2	64.7%	62.9%	71.8%	2.7	2.6	4.1	4.2	4.0	6.3	85.0%	65.0%	75.0%
2016-2019	2021-Q3	65.0%	64.3%	72.6%	2.7	2.7	4.1	4.5	4.2	6.8	75.0%	45.0%	65.0%
2016-2019	2021-Q4	66.2%	60.4%	73.7%	2.3	2.7	3.8	3.8	4.4	5.6	65.0%	70.0%	70.0%
**Univariate Model**													
2016-2020	2021-Ann	68.2%	67.0%	71.2%	9.8	8.1	15.4	16.7	15.2	26.7	60.0%	60.0%	55.0%
2016-2019	2021-Ann	68.1%	65.4%	69.1%	10.8	8.6	17.0	22.5	15.3	31.3	60.0%	60.0%	55.0%
2016-2020	2021-Q1	59.3%	58.8%	68.4%	2.8	2.8	2.8	4.9	4.1	7.7	75.0%	50.0%	65.0%
2016-2020	2021-Q2	63.3%	58.0%	66.5%	2.7	2.7	2.7	4.8	4.7	8.2	75.0%	50.0%	60.0%
2016-2020	2021-Q3	61.4%	58.0%	67.8%	4.6	4.6	4.6	5.4	4.2	8.7	70.0%	60.0%	60.0%
2016-2020	2021-Q4	60.1%	55.9%	64.1%	3.0	3.0	3.0	5.2	4.3	7.6	70.0%	60.0%	60.0%
2016-2019	2021-Q1	59.7%	59.5%	67.1%	2.9	2.9	2.9	5.5	4.3	8.6	70.0%	50.0%	60.0%
2016-2019	2021-Q2	62.9%	60.0%	69.1%	5.1	5.1	5.1	5.0	4.7	8.7	75.0%	55.0%	55.0%
2016-2019	2021-Q3	61.7%	59.4%	67.7%	3.2	3.2	3.2	5.9	4.5	9.0	65.0%	55.0%	50.0%
2016-2019	2021-Q4	59.8%	59.0%	66.0%	2.7	2.7	2.7	5.5	4.7	8.5	65.0%	55.0%	55.0%

**Previous Hospitalizations**													
2020	2021-Ann	75.0%	70.3%	80.8%	5.0	5.2	7.6	7.4	7.8	11.4	85.0%	75.0%	75.0%
2019	2021-Ann	74.3%	66.7%	75.6%	5.7	7.0	9.7	9.3	10.5	15.4	85.0%	75.0%	75.0%
2020	2021-Q1	66.7%	63.6%	72.7%	2.0	2.0	2.0	2.9	2.9	4.2	80.0%	70.0%	75.0%
2020	2021-Q2	66.7%	62.5%	71.5%	2.1	2.1	2.1	3.6	3.7	5.5	85.0%	60.0%	70.0%
2020	2021-Q3	66.7%	57.1%	69.4%	2.9	2.9	2.9	3.8	4.1	5.8	75.0%	55.0%	70.0%
2020	2021-Q4	66.7%	55.6%	66.7%	2.3	2.3	2.3	3.2	3.9	5.5	70.0%	60.0%	80.0%
2019	2021-Q1	58.3%	58.7%	69.6%	2.4	2.4	2.4	3.3	3.7	5.5	80.0%	75.0%	85.0%
2019	2021-Q2	61.5%	58.3%	66.7%	3.6	3.6	3.6	3.7	4.3	5.9	80.0%	65.0%	85.0%
2019	2021-Q3	57.3%	50.0%	64.1%	2.4	2.4	2.4	4.0	5.0	6.8	70.0%	55.0%	70.0%
2019	2021-Q4	60.0%	50.0%	62.5%	2.6	2.6	2.6	3.9	4.9	7.1	70.0%	60.0%	70.0%

Main columns show the median agreement percentage (MAP), mean absolute error (MAE), root mean squared error (RMSE) of hospitalizations counts in all ZCTAs, and ranking agreement percentage (RAP) of top 20 ZCTAs in South Carolina. These metrics are calculated for opioid, HCV and combined hospitalizations using fully adjusted (includes all variables in the model) and univariate (only includes lagged hospitalization counts in the model) negative Binomial, Poisson and conditional autoregressive (CAR) models. The benchmark method utilizes previous year hospitalizations and is added in the bottom panel. Results are summarized both annual and quarterly data based on different training data settings (2016 to 2020 and 2016 to 2019 for regression models, and 2020 and 2019 for the benchmark method).

8. Results for Interrupted Times Series

Table A14: Interrupted time series (Negative Binomial generalized linear mixed effects) model results for opioid, HCV, and combined hospitalizations adjusted for time where the population is an offset.

Variable	Opioid Hospitalizations			HCV Hospitalizations			Combined Hospitalizations		
	RR	CI	p-value	RR	CI	p-value	RR	CI	p-value
**Age** (Ref: % Age 18-29)									
% Age 30-44	1.01	0.93 - 1.1	0.78	0.89	0.82 - 0.97	**0.006**	0.96	0.88 - 1.04	0.29
% Age 45-64	1.11	1.03 - 1.21	0.008	1.08	1.00 - 1.18	**0.047**	1.10	1.02 - 1.19	**0.012**
% Age above 65	1.05	0.96 - 1.14	0.28	0.84	0.78 - 0.91	**<0.0001**	0.96	0.89 - 1.04	0.36
**Sex** (Ref: % Female)									
% Male	0.97	0.91 - 1.04	0.41	0.98	0.9 - 1.07	0.72	0.96	0.9 - 1.02	0.20
**Race** (Ref: % White)									
% Black	0.99	0.92 - 1.05	0.66	0.99	0.92 - 1.06	0.73	0.98	0.92 - 1.04	0.50
% Other race	0.97	0.91 - 1.03	0.35	1.03	0.96 - 1.11	0.42	0.98	0.92 - 1.05	0.59
**Ethnicity** (Ref: % Non-Hispanic)									
% Hispanic	0.99	0.93 - 1.06	0.86	1.00	0.93 - 1.07	0.97	1.00	0.94 - 1.07	0.89
**Other**									
Time (year)$^{\#}$	1.00	0.99 - 1.00	0.13	1.00	0.99 - 1	**0.035**	1.00	0.99 - 1.00	**0.043**
Interruption	0.97	0.90 - 1.05	0.46	0.93	0.86 - 1	**0.038**	0.95	0.89 - 1.01	0.12
Interruption*Time	1.01	0.99 - 1.03	0.20	0.96	0.95 - 0.98	**<0.0001**	0.99	0.98 - 1.01	0.22
LH	1.08	1.06 - 1.09	**<0.0001**	1.11	1.09 - 1.12	**<0.0001**	1.08	1.07 - 1.1	**<0.0001**
CLOH	-	-	**-**	1.00	1.00 - 1.00	0.085	-	-	**-**
**Socioeconomic**									
SVI	1.18	1.12 - 1.26	**<0.0001**	1.18	1.1 - 1.26	**<0.0001**	1.17	1.11 - 1.24	**<0.0001**
Median Income	0.78	0.73 - 0.82	**<0.0001**	0.8	0.75 - 0.85	**<0.0001**	0.79	0.75 - 0.83	**<0.0001**
% in Poverty	1.24	1.16 - 1.31	**<0.0001**	1.24	1.16 - 1.32	**<0.0001**	1.25	1.18 - 1.32	**<0.0001**
% Unemployed	0.91	0.85 - 0.97	**0.0052**	0.93	0.87 - 1	**0.042**	0.87	0.82 - 0.93	**<0.0001**
% Labor Force Participation	1.12	1.05 - 1.20	**0.0004**	0.9	0.84 - 0.97	**0.0077**	1.01	0.94 - 1.08	0.81
% Rural	1.10	1.04 - 1.17	**0.0016**	1.01	0.95 - 1.09	0.71	1.05	0.98 - 1.11	0.14
**Healthcare access**									
% Uninsured	1.24	1.17 - 1.32	**<0.0001**	1.19	1.11 - 1.28	**<0.0001**	1.24	1.17 - 1.32	**<0.0001**

Variable	Opioid Hospitalizations			HCV Hospitalizations			Combined Hospitalizations		
	RR	CI	p-value	RR	CI	p-value	RR	CI	p-value
PCP Rate per 1000	1.02	0.94 - 1.09	0.68	1.08	1.00 - 1.16	**0.036**	1.05	0.99 - 1.12	0.10
MD/DO Rate per 1000	1.02	0.95 - 1.09	0.63	1.08	1.01 - 1.16	**0.035**	1.06	0.99 - 1.13	0.097
Hospital Present in ZCTA	1.09	0.93 - 1.27	0.28	1.09	0.92 - 1.30	0.33	1.13	0.97 - 1.33	0.12
**Mortality Rate per 1000**	1.21	1.14 - 1.28	**<0.0001**	1.17	1.10 - 1.25	**<0.0001**	1.18	1.11 - 1.25	**<0.0001**

Estimated relative risk (RR) represents exponentiated regression coefficients. Associated 95% confidence intervals (CI) and p-values for each RR are provided. The RR are interpreted as the relative change in ZCTA-level hospitalization risk for a standard deviation increase in the predictor variable. HCV: hepatitis C virus, SVI: social vulnerability index, PCP: primary care physicians MD/DO: medical doctors or doctor of osteopathic medicine, LH: Lagged hospitalization counts, CLOH: Cumulative lagged opioid hospitalization counts, and Ref indicates the reference group. **Bolded p-values** represent statistical significance (P<0.05).

[#] Variable adjusted for in all models.

Table A15: Results for the interrupted time series (Negative Binomial) model. Columns show the median agreement percentage (MAP), and ranking agreement percentage (RAP) of top 20 ZCTAs using training data of 2016-2020 and validation data of 2021.

		MAP			RAP		
Training Data	Validation Data	A_{OP}	A_{HCV}	A_{Comb}	A_{OP}	A_{HCV}	A_{Comb}
2016-2020	2021-Q1	66.1%	67.5%	76.3%	85.0%	70.0%	80.0%
2016-2020	2021-Q2	68.2%	62.1%	73.4%	90.0%	65.0%	85.0%
2016-2020	2021-Q3	71.6%	61.7%	74.2%	75.0%	55.0%	70.0%
2016-2020	2021-Q4	66.0%	58.7%	71.0%	65.0%	60.0%	70.0%

Figure A3: Quarterly hospitalization counts for opioid and HCV per ZCTA and overall SC from 2016 to 2021.

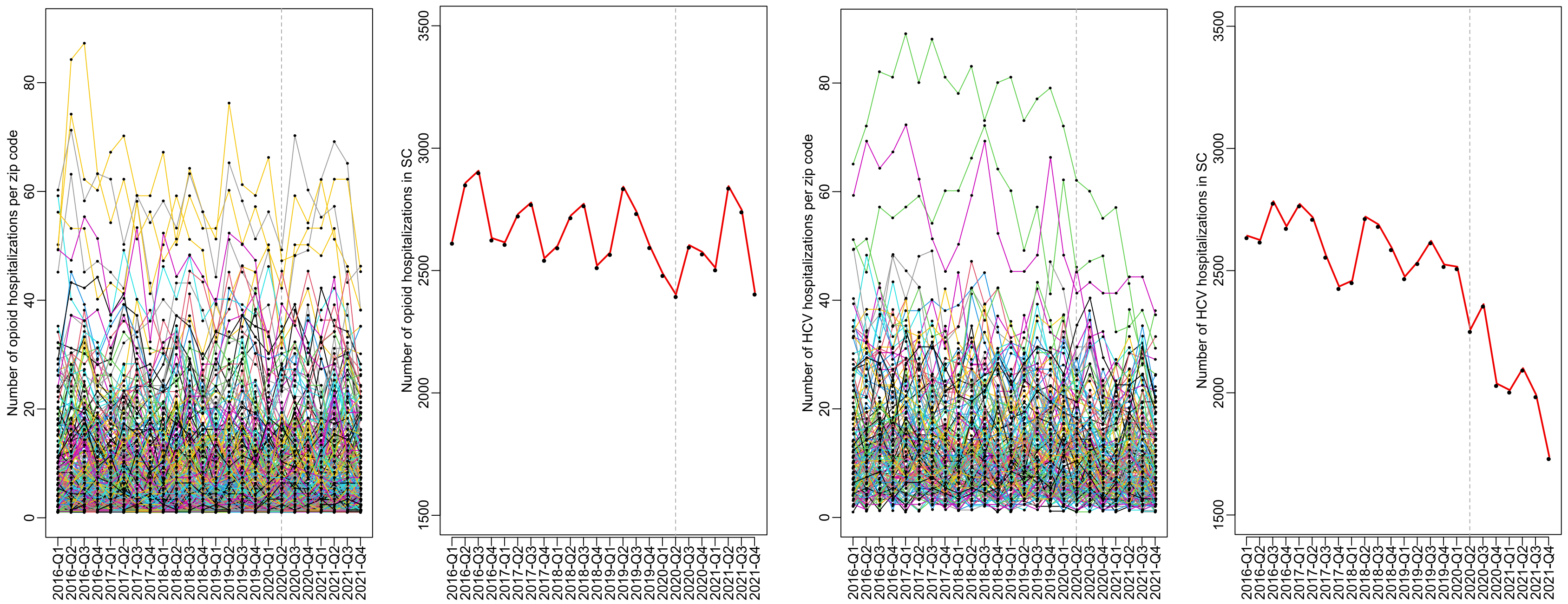

Figure A1: South Carolina County map.

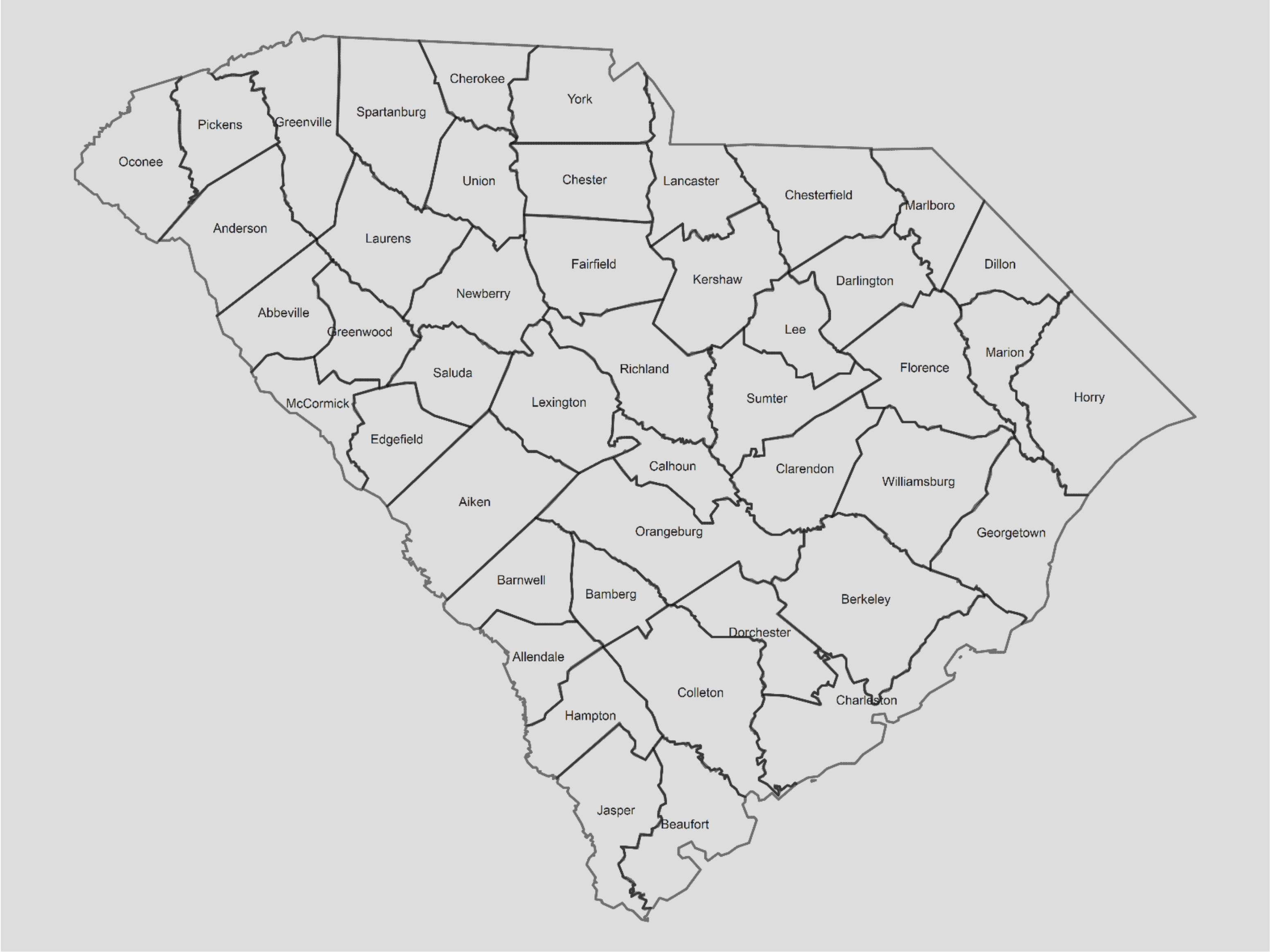

Figure A2: Predicted opioid (top) and HCV (bottom) hospitalizations across the top 20 of highest-risk ZCTAs in Upstate and Midlands regions of SC in 2023. Locations of the healthcare facilities in these ZCTAs are marked based on the facility type. ZCTAs that are in the top quartile of SVI and uninsured rates are hatched with black and dark red lines, respectively.

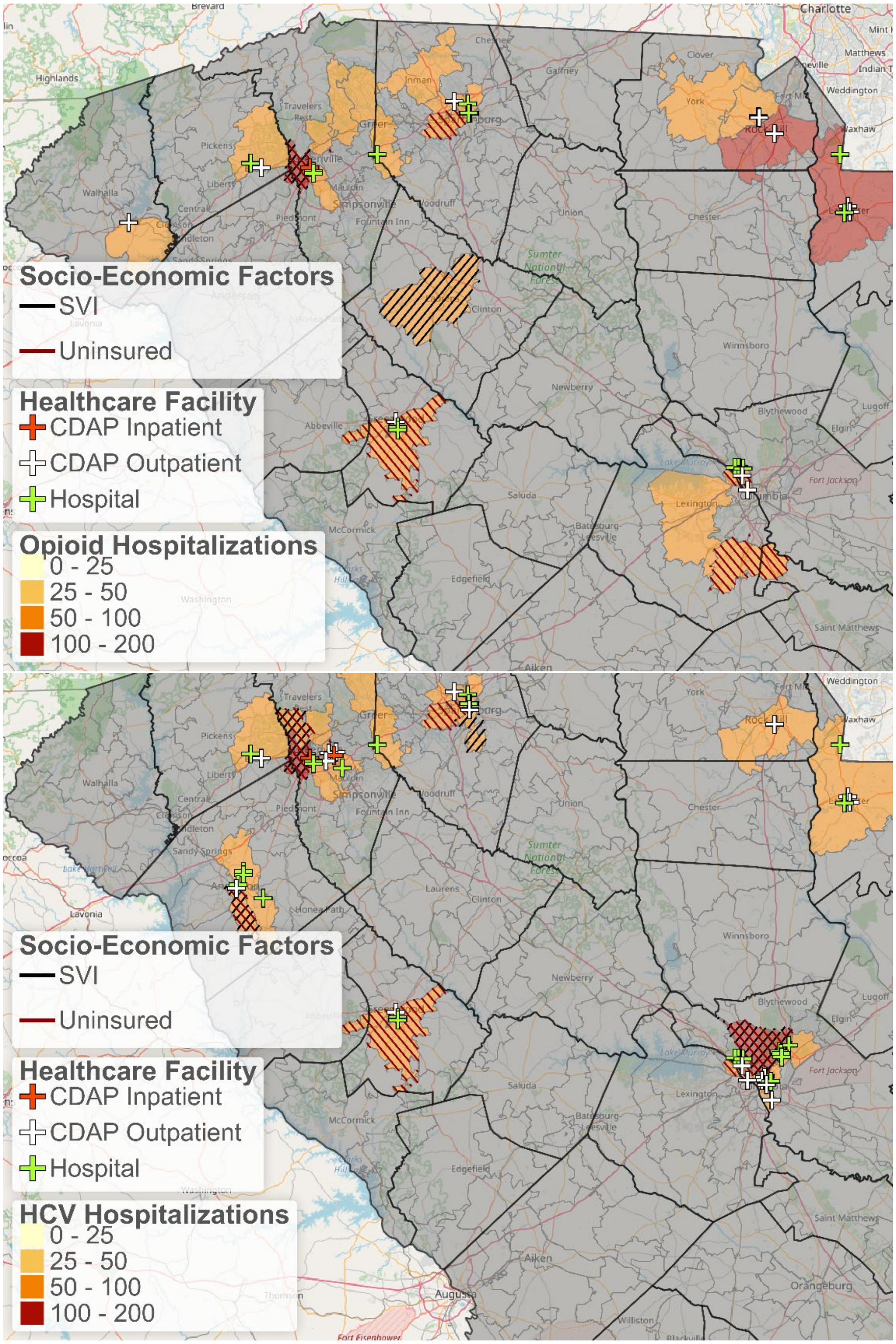

9. Characteristics of High-Risk ZCTAs

Table A16: Characteristics of ZCTAs with the highest number of predicted opioid-related hospitalizations in the Upstate and Midlands regions in SC.

ZCTA	County	Pred. Hosp.	Prev. Hosp.	SVI	% of Uninsured	Median Income	% in Poverty	# of Hosp.	% Hosp. > 30	MD/DO Rate	PCP Rate
29730	York	120	143	0.5	11.2	48,208	11.8	0	100	0.0	0.0
29720	Lancaster	103	111	0.6	11.8	42,927	14.5	2	95.6	0.9	0.5
29611	Greenville	101	94	0.7	19.2	35,267	24.1	0	100	0.2	0.1
29640	Pickens	87	82	0.5	11.1	48,611	11.4	1	100	3.1	2.2
29732	York	84	98	0.4	7.5	64,342	5.9	2	100	0.0	0.0
29646	Greenwood	82	84	0.6	14.6	34,656	22.1	2	98	0.8	0.6
29651	Greenville	77	79	0.4	11.6	60,646	6.7	1	100	0.0	0.0
29073	Lexington	74	82	0.4	11.1	60,521	11.4	0	100	0.0	0.0
29605	Greenville	71	54	0.6	11.2	51,519	14.6	2	100	3.4	2.4
29303	Spartanburg	66	66	0.7	11.9	36,450	18.7	3	100	2.3	1.8
29301	Spartanburg	63	58	0.6	12.9	45,998	14.8	0	100	0.0	0.0
29687	Greenville	61	49	0.3	10.6	59,663	6	0	100	0.5	0.3
29360	Laurens	58	53	0.6	11.5	41,567	17.1	0	97.3	2.7	2.3
29745	York	58	55	0.3	9.7	55,536	12.5	0	89.2	1.0	0.7
29609	Greenville	57	59	0.5	14.1	55,077	11	0	100	0.1	0.1
29210	Richland	57	62	0.4	12.6	41,345	14.5	4	100	15.1	9.5
29053	Lexington	57	56	0.6	18	41,481	21.2	0	95	0.0	0.0
29678	Oconee	56	42	0.5	11.3	46,130	15.2	0	100	0.0	0.0
29349	Spartanburg	54	44	0.4	9.1	61,503	7.5	0	100	0.1	0.0
29072	Lexington	54	63	0.3	4.2	87,667	4.5	0	77.4	0.2	0.1

Table A17: Characteristics of ZCTAs with the highest number of predicted HCV-related hospitalizations in the Upstate and Midlands regions in SC.

ZCTA	County	Pred. Hosp.	Prev. Hosp.	SVI	% of Uninsured	Median Income	% in Poverty	# of Hosp.	% Hosp. > 30	MD/DO Rate	PCP Rate
29203	Richland	155	179	0.8	13.3	30,922	30.6	6	100.0	4.1	2.6
29611	Greenville	125	135	0.7	19.2	35,267	24.1	0	100.0	0.2	0.1
29210	Richland	85	104	0.4	12.6	41,345	14.5	4	100.0	15.1	9.5
29605	Greenville	82	76	0.6	11.2	51,519	14.6	2	100.0	3.4	2.4
29303	Spartanburg	77	102	0.7	11.9	36,450	18.7	3	100.0	2.3	1.8
29651	Greenville	67	86	0.4	11.6	60,646	6.7	1	100.0	0.0	0.0
29730	York	67	93	0.5	11.2	48,208	11.8	0	100.0	0.0	0.0
29204	Richland	66	79	0.5	12.4	42,826	14.1	1	100.0	3.2	1.8
29640	Pickens	66	76	0.5	11.1	48,611	11.4	1	100.0	3.1	2.2
29607	Greenville	66	73	0.3	9.5	60,823	8.4	0	100.0	1.1	0.7
29609	Greenville	65	76	0.5	14.1	55,077	11.0	0	100.0	0.1	0.1
29720	Lancaster	65	79	0.6	11.8	42,927	14.5	2	95.6	0.9	0.5
29306	Spartanburg	64	80	0.7	10.7	32,824	24.2	0	100.0	0.0	0.0
29223	Richland	63	71	0.6	12.0	50,258	13.7	0	100.0	2.9	2.1
29201	Richland	62	74	0.4	7.1	31,160	16.7	0	100.0	1.9	0.9
29624	Anderson	60	66	0.8	16.1	27,107	24.7	0	100.0	0.0	0.0
29301	Spartanburg	59	77	0.6	12.9	45,998	14.8	0	100.0	0.0	0.0
29617	Greenville	57	59	0.8	21.4	40,793	14.1	0	100.0	0.0	0.0
29621	Anderson	57	61	0.5	5.8	61,599	7.9	4	100.0	0.5	0.3
29646	Greenwood	57	81	0.6	14.6	34,656	22.1	2	98.0	0.8	0.6

Table A18: Characteristics of ZCTAs with the highest number of predicted combined hospitalizations in the Upstate and Midlands regions in SC.

ZCTA	County	Pred. Hosp.	Prev. Hosp.	SVI	% of Uninsured	Median Income	% in Poverty	# of Hosp.	% Hosp. > 30	MD/DO Rate	PCP Rate
29611	Greenville	236	229	0.7	19.2	35,267	24.1	0	100.0	0.2	0.1
29203	Richland	187	220	0.8	13.3	30,922	30.6	6	100.0	4.1	2.6
29640	Pickens	158	158	0.5	11.1	48,611	11.4	1	100.0	3.1	2.2
29646	Greenwood	152	165	0.6	14.6	34,656	22.1	2	98.0	0.8	0.6
29720	Lancaster	150	190	0.6	11.8	42,927	14.5	2	95.6	0.9	0.5
29303	Spartanburg	147	168	0.7	11.9	36,450	18.7	3	100.0	2.3	1.8
29651	Greenville	145	165	0.4	11.6	60,646	6.7	1	100.0	0.0	0.0
29210	Richland	136	166	0.4	12.6	41,345	14.5	4	100.0	15.1	9.5
29732	York	126	152	0.4	7.5	64,342	5.9	2	100.0	0.0	0.0
29607	Greenville	125	121	0.3	9.5	60,823	8.4	0	100.0	1.1	0.7
29301	Spartanburg	124	135	0.6	12.9	45,998	14.8	0	100.0	0.0	0.0
29609	Greenville	122	135	0.5	14.1	55,077	11.0	0	100.0	0.1	0.1
29360	Laurens	119	107	0.6	11.5	41,567	17.1	0	97.3	2.7	2.3
29073	Lexington	117	132	0.4	11.1	60,521	11.4	0	100.0	0.0	0.0
29687	Greenville	116	106	0.3	10.6	59,663	6.0	0	100.0	0.5	0.3
29223	Richland	112	109	0.6	12.0	50,258	13.7	0	100.0	2.9	2.1
29624	Anderson	110	112	0.8	16.1	27,107	24.7	0	100.0	0.0	0.0
29611	Greenville	236	229	0.7	19.2	35,267	24.1	0	100.0	0.2	0.1
29203	Richland	187	220	0.8	13.3	30,922	30.6	6	100.0	4.1	2.6
29640	Pickens	158	158	0.5	11.1	48,611	11.4	1	100.0	3.1	2.2

10. County Level Risk Assessment

Table A19: Summary statistics of counties with highest risk opioid, HCV and combined hospitalizations.

County	ZCTAs	Counts	Median (IQR)	Mean (SD)
Opioid Hospitalizations				
Horry County	16	1,271	62 (32 - 107)	79 (62)
Florence County	11	449	33 (18 - 47)	41 (36)
Richland County	15	429	27 (16 - 37)	29 (17)
Berkeley County	13	418	12 (5 - 43)	32 (41)
Lexington County	13	404	21 (15 - 49)	31 (21)
York County	11	343	11 (4 - 44)	31 (40)
Anderson County	12	294	21 (15 - 31)	25 (14)
Georgetown County	3	282	68 (53 - 123)	94 (74)
Pickens County	9	243	16 (6 - 39)	27 (29)
Beaufort County	11	229	20 (11 - 28)	21 (14)
HCV Hospitalizations				
Richland County	15	660	30 (18 - 63)	44 (39)
Horry County	16	490	28 (14 - 36)	31 (24)
Anderson County	12	330	26 (13 - 34)	27 (19)
Lexington County	13	269	13 (11 - 30)	21 (13)
Pickens County	9	186	12 (5 - 28)	21 (22)
Berkeley County	12	181	6 (4 - 29)	15 (17)
York County	11	167	4 (2 - 20)	15 (21)
Florence County	11	167	10 (6 - 18)	15 (16)
Greenwood County	6	125	10 (9 - 31)	21 (21)
Beaufort County	10	116	10 (7 - 15)	12 (6)
Combined Hospitalizations				
Horry County	16	1,741	89 (48 - 143)	109 (84)
Richland County	15	1,059	59 (32 - 95)	71 (49)

Anderson County	12	681	50 (29 - 69)	57 (39)
Lexington County	13	656	33 (27 - 80)	50 (32)
Florence County	11	596	44 (23 - 64)	54 (52)
Berkeley County	13	594	18 (6 - 67)	46 (56)
York County	11	563	15 (6 - 63)	51 (73)
Pickens County	9	436	28 (12 - 68)	48 (52)
Beaufort County	11	358	30 (18 - 47)	33 (20)
Georgetown County	3	345	84 (66 - 148)	115 (86)

Table A20: The median agreement percentage (MAP), mean absolute error (MAE) and root mean squared error (RMSE) of the county-level hospitalization rate and ZCTA-level hospitalization rate within the same county in 2021. The county-level hospitalization rate, range (min, max) of ZCTA hospitalization rate (within county), and MAE and RMSE are multiplied by 1000 for each hospitalization type.

	Opioid Hospitalizations					HCV Hospitalizations					Combined Hospitalizations				
**County**	**Rate**	**ZCTA range**	**MAP**	**MAE**	**RMSE**	**Rate**	**ZCTA range**	**MAP**	**MAE**	**RMSE**	**Rate**	**ZCTA range**	**MAP**	**MAE**	**RMSE**
Abbeville	0.8	(0.3 - 1.2)	71.3	0.3	0.3	0.9	(0.6 - 11.2)	72.2	2.2	4.6	1.7	(0.9 - 2)	87.5	0.3	0.4
Aiken	1.1	(0 - 4.4)	69.1	0.7	1	0.6	(0 - 1.8)	56.3	0.4	0.5	1.7	(0 - 5.8)	67	1	1.4
Allendale	1	(0.7 - 1.2)	78.4	0.2	0.2	0.2	(0.2 - 0.2)	98.8	0	0	1.2	(1 - 1.4)	82.6	0.2	0.2
Anderson	1.6	(1 - 2.6)	75.4	0.4	0.5	2.3	(1.3 - 5.3)	76.3	0.8	1.1	3.9	(2.3 - 7.5)	82.4	1.1	1.4
Bamberg	1.3	(1.1 - 2.5)	69.5	0.6	0.8	0.7	(0 - 1.1)	56	0.5	0.5	2	(1.6 - 2.5)	83.3	0.3	0.4
Barnwell	1.9	(1.7 - 2.1)	91.7	0.2	0.2	0.6	(0.2 - 0.8)	73.9	0.2	0.3	2.5	(1.9 - 2.9)	86.9	0.4	0.4
Beaufort	1.4	(0.6 - 3.4)	60.8	0.7	0.9	0.6	(0.3 - 2)	64.1	0.4	0.5	2	(1 - 4.8)	67.9	0.9	1.2
Berkeley	2.2	(0.9 - 6.6)	73.2	1.2	1.7	1	(0.1 - 2.4)	65.5	0.6	0.8	3.3	(1 - 8.8)	69.2	1.7	2.3
Calhoun	1.6	(1.4 - 2.6)	75.2	0.6	0.7	0.6	(0.6 - 0.6)	100	0	0	2.1	(2 - 2.6)	88.6	0.3	0.3
Charleston	1.9	(0 - 3.7)	61.3	0.9	1.1	1.5	(0 - 3.3)	72.7	0.7	0.9	3.4	(0 - 6.7)	67.9	1.6	1.9
Cherokee	1.7	(1.3 - 2.5)	76	0.4	0.5	1.7	(1.1 - 2.3)	74.2	0.4	0.5	3.4	(2.3 - 4.3)	78.8	0.9	0.9
Chester	2.2	(1.6 - 4)	80.9	0.7	0.9	1.2	(0.7 - 1.5)	81.7	0.2	0.3	3.3	(2.8 - 5.1)	88.3	0.7	1
Chesterfield	1.2	(0.3 - 2.5)	83.8	0.5	0.7	0.6	(0 - 1.3)	75	0.3	0.4	1.8	(0.3 - 3.5)	72	0.8	1
Clarendon	1.7	(1.2 - 2.6)	70	0.5	0.6	0.9	(0 - 2)	66.2	0.6	0.7	2.6	(2.3 - 4.6)	93.8	0.5	0.9
Colleton	2.5	(1 - 5.5)	72.7	1	1.3	1	(0.6 - 2.2)	68	0.5	0.7	3.4	(1 - 7.7)	64.2	1.6	1.9
Darlington	3.1	(1.8 - 3.3)	89.4	0.6	0.7	1.2	(0.2 - 1.7)	70.3	0.5	0.6	4.3	(2.4 - 5)	86.7	0.9	1.2
Dillon	3.3	(1.7 - 4.6)	80.8	0.9	1.1	1.2	(0 - 3.4)	74	0.8	1.1	4.5	(1.7 - 5.9)	83.7	1.2	1.4
Dorchester	1.7	(1.3 - 3.5)	85.2	0.5	0.8	0.8	(0.3 - 1.7)	80.7	0.3	0.4	2.5	(2.2 - 5.2)	84.8	0.7	1.1
Edgefield	0.6	(0.2 - 1.1)	72.6	0.3	0.3	0.4	(0 - 1.2)	61.7	0.3	0.5	1	(0.6 - 1.9)	55.2	0.6	0.6
Fairfield	2.4	(1.2 - 2.6)	92	0.5	0.7	0.9	(0 - 1.4)	0	0.8	0.8	3.3	(1.2 - 3.9)	78.5	1.1	1.3
Florence	3.1	(2.2 - 8.1)	89.2	1	1.7	1.2	(0 - 2.4)	73.3	0.5	0.6	4.3	(2.3 - 10.5)	91.5	1.2	2.1
Georgetown	5.5	(2.4 - 6.8)	81.1	1.9	2	1.5	(0.9 - 1.8)	81.7	0.4	0.4	7	(3.3 - 8.6)	81.2	2.2	2.5
Greenville	1.7	(0.9 - 4.9)	77.5	0.7	1.1	1.8	(0.8 - 14.6)	72.5	1.5	3.2	3.4	(1.7 - 19.5)	79.4	2.1	4.1
Greenwood	2.4	(1.6 - 2.9)	82	0.5	0.5	2.4	(1.8 - 2.9)	80.7	0.5	0.5	4.7	(1.6 - 5.8)	93.1	0.9	1.3
Hampton	2.6	(1.2 - 5.2)	58.1	1.2	1.5	0.7	(0 - 2)	45	0.5	0.7	3.3	(1.2 - 7.2)	68.5	1.4	1.9
Horry	4	(2.8 - 9)	79.1	1.3	1.9	1.7	(0.7 - 3.6)	80.5	0.6	0.8	5.6	(3.9 - 11.8)	75.4	1.8	2.5
Jasper	2.2	(1.3 - 2.8)	77.8	0.6	0.6	1.1	(0.9 - 1.2)	86.5	0.2	0.2	3.2	(1.7 - 4)	70.5	1.1	1.1

Kershaw	2.1	(1.4 - 17.9)	71.7	3.3	6.5	1.1	(0.2 - 8.9)	73.1	1.6	3.2	3.2	(2.3 - 26.8)	77	4.7	9.7
Lancaster	1.3	(0.2 - 1.9)	68	0.7	0.7	0.7	(0.1 - 1.1)	69.5	0.4	0.4	2	(0.3 - 3)	70.6	1.1	1.1
Laurens	3	(0 - 3.9)	77.7	1.1	1.5	2.9	(0.8 - 4.5)	76.5	1	1.1	5.9	(0.8 - 7.5)	81.6	1.8	2.4
Lee	1.9	(1 - 2.1)	73.1	0.5	0.6	0.7	(0 - 0.8)	40.7	0.4	0.5	2.5	(1 - 2.9)	64.7	0.9	1.1
Lexington	1.4	(0.7 - 2.6)	78.8	0.4	0.6	0.8	(0.3 - 1.7)	62.9	0.4	0.5	2.2	(1.1 - 4.2)	74.8	0.8	1
Marion	2.6	(2 - 5)	83.5	0.9	1.3	1.3	(0.3 - 1.6)	87.8	0.3	0.5	3.8	(3 - 6.2)	85.9	0.9	1.3
Marlboro	1.1	(0.9 - 1.8)	82.9	0.3	0.3	0.9	(0.8 - 2.3)	89.5	0.4	0.7	2	(1 - 3.7)	76.9	0.8	0.9
McCormick	0.6	(0.3 - 2)	42.9	0.9	1.1	0.2	(0.2 - 0.2)	100	0	0	0.7	(0.5 - 2)	52.1	0.8	1
Newberry	1.1	(0 - 2.2)	62.9	0.7	0.8	0.6	(0 - 3.3)	59.5	0.6	1.1	1.6	(1 - 3.3)	60	0.8	1
Oconee	2.5	(0 - 3.4)	84.3	0.7	1	2.4	(1 - 5.3)	71.9	0.9	1.2	4.9	(2.9 - 5.5)	88.8	0.8	1
Orangeburg	1.2	(0 - 2.7)	72.6	0.5	0.6	0.6	(0 - 3.3)	67	0.5	0.8	1.8	(0 - 4.4)	71.9	0.8	1.1
Pickens	2	(0.3 - 3.8)	56.8	1.1	1.2	1.6	(0 - 3.8)	63.6	0.8	1.1	3.6	(0.6 - 7.6)	64.4	1.8	2.2
Richland	1	(0 - 1.7)	71.4	0.3	0.4	1.4	(0 - 3.4)	48.8	0.8	1	2.4	(0 - 4.6)	58.2	1	1.3
Saluda	0.7	(0 - 1.1)	70.3	0.4	0.5	0.5	(0.4 - 0.7)	80.4	0.1	0.1	1.2	(0 - 1.8)	69	0.6	0.8
Spartanburg	1.9	(0 - 76.9)	68.1	3.9	14	1.7	(0 - 22.9)	63.3	2	4.6	3.6	(0 - 76.9)	66.6	5.6	14.7
Sumter	1.3	(0 - 1.5)	82	0.5	0.7	0.9	(0 - 2.2)	75.8	0.4	0.6	2.1	(0 - 3.6)	57.8	1	1.2
Union	2.3	(1.8 - 3.5)	89	0.4	0.6	1.1	(0 - 1.8)	61.9	0.8	0.8	3.4	(2.2 - 5.2)	76.6	0.9	1
Williamsburg	2.7	(0 - 4.9)	55.1	1.5	1.7	1.1	(0.6 - 2.9)	50.8	0.8	1	3.7	(0 - 6.3)	71.1	1.6	2.1
York	1.3	(0.1 - 2.6)	55.7	0.8	0.9	0.8	(0 - 1.9)	55.4	0.6	0.6	2.1	(0.2 - 4.5)	61.8	1.1	1.3
Median (IQR)	1.8 (1.3-2.4)		75.3 (69.6 - 81.8)	0.7 (0.5-0.9)	0.8 (0.6-1.2)	1.0 (0.7-1.4)		72.4 (63.0-70.4)	0.5 (0.4-0.8)	0.6 (0.5-1.0)	3.2 (2.0-3.6)		76.0 (67.9-83.6)	0.9 (0.8-1.4)	1.2 (1.0-1.9)
Mean (SD)	1.9 (0.9)		74.4 (10.6)	0.8 (0.7)	1.3 (2.1)	1.1 (0.6)		69.7 (16.9)	0.6 (0.5)	0.9 (1.0)	3.0 (1.3)		75.2 (10.8)	1.2 (1.0)	1.8 (2.4)

11. Code

All analysis in this manuscript and are performed using R version 4.3.1 programming language. Following packages and functions are used for the fully adjusted regression models. Hence the formulation for the models is given in Section 1.1 for generalized linear mixed effects model and in Section 1.2 for conditionally autoregressive (CAR) model in of this appendix. Variables and corresponding labels in the model are provided in **Table A21.**

Table A21: Variable labels for code expression

Variable	Label
Hospitalization counts (Response variable)	counts
% Age 30-44	age.30.44
% Age 45-64	age.45.64
% Age above 65	age.65.over
% Male	male
% Black	black
% Other race	other.race
% Hispanic	hispanic
Time	time
Lag Counts	lag.counts
SVI	SVI
% Uninsured	uninsured
Median Income	income
% in Poverty	poverty
% Unemployed	unemployed
% Labor Force Participation	labor
% Rural	rural
PCP Rate	PCP
MD/DO Rate*	MD/DO
Hospital Present	hospital
Mortality Rate	mortality

*Either MD/DO rate or PCP rate is included in the fully adjusted models.

Negative Binomial generalized linear mixed effects model (Table A6):

```
nb.mod = lme4::glmer.nb(counts ~ time + lag.counts +
    age.30.44 + age.45.64 + age.65.over + male + black + other.race + hispanic +
    SVI + income + rural + poverty + unemployed + labor + PCP +   hospital +
    uninsured + mortality + offset(log(population)) + (1|zcta) + (1|time)*time,
    data=analysis.data)
```

Poisson generalized linear mixed effects model (Table A10):

```
pois.mod = lme4::glmer(counts ~ time + lag.counts +
    age.30.44 + age.45.64 + age.65.over + male + black + other.race + hispanic +
    SVI + income + rural + poverty + unemployed + labor + PCP +   hospital +
    uninsured + mortality + offset(log(population)) + (1|zcta) + (1|time)*time,
    family = 'poisson' data=analysis.data)
```

Conditionally Autoregressive (CAR) model (Table A12):

```
# Model parameter settings

Ncar = 50000; burn.in.car = 10000; thin = 10

# W denotes the neighborhood matrix

car.mod = lme4::glmer(counts ~ lag.counts +
    age.30.44 + age.45.64 + age.65.over + male + black + other.Race + Hispanic +
    SVI + income + rural + poverty + unemployed + labor + PCP +   hospital +
    uninsured + mortality + offset(log(population), scol = "zcta",
    tcol = "qtime", W=nbs.mat, model="ar", AR=1, family="poisson",
    package="CARBayesST", N=Ncar, burn.in=burn.in.car, thin=thin, interaction =
    T, verbose=Fdata=analysis.data)
```

References

1. Bauer C, Zhang K, Li W, Bernson D, Dammann O, LaRochelle MR, et al. Small Area Forecasting of Opioid-Related Mortality: Bayesian Spatiotemporal Dynamic Modeling Approach. JMIR Public Health Surveill. 2023 Feb 10;9(1):e41450.

2. Held HR Leonhard. Gaussian Markov Random Fields: Theory and Applications. New York: Chapman and Hall/CRC; 2005. 280 p.

3. Besag J. Spatial Interaction and the Statistical Analysis of Lattice Systems. J R Stat Soc Ser B Methodol. 1974;36(2):192–225.

4. Miles J. Tolerance and Variance Inflation Factor. In: Encyclopedia of Statistics in Behavioral Science [Internet]. John Wiley & Sons, Ltd; 2005 [cited 2023 Nov 24]. Available from: https://onlinelibrary.wiley.com/doi/abs/10.1002/0470013192.bsa683
